# NIR and Oxidative Stress-Modulated Intestinal Peristalsis Motion of Tubular Conductive Thermo-Hydrogel Actuator

**DOI:** 10.1007/s40820-026-02257-x

**Published:** 2026-07-06

**Authors:** A. Hyeon Kim, Hayeon Jo, Kaustuv Roy, Sung Young Park

**Affiliations:** 1 Department of IT and Energy Convergence, Korea National University of Transportation, Chungju, 27469 Republic of Korea; 2 School of Nanomedical Engineering, Korea National University of Transportation, Chungju, 27469 Republic of Korea

**Keywords:** NIR- and ROS-responsive, Intestinal peristalsis, Thermo-hydrogel actuator, Fluorescence on/off, ROS-scavenging

## Abstract

Actuators for soft bionic robotics, utilizing disease microenvironment-responsive and mechanically flexible materials, are influenced by intra- and intermolecular structures of hydrogels. However, they lack the required endogenous stimuli responsiveness necessary for applications like gastrointestinal-targeted drug screening and liquid/mass transport. Herein, a tubular conductive, reactive oxygen species (ROS)- and an near infrared (NIR)-responsive thermo-hydrogel actuator (THA) was fabricated for viscous liquid and solid transport. The presence of diselenide-crosslinked polymer dots (PDs) loaded onto polydopamine (PDA) nanoparticles (PD@PDA) in a poly(*N*-isopropylacrylamide) and polyvinyl alcohol (PNIPAM/PVA) matrix provided the necessary LCST alongside ROS- and NIR-responsive behavior. In the presence of high ROS, the diselenide bonds cleaved, resulting in release of PDA, generating NIR-specific actuation. The actuation process was confirmed by the directional movement of silicone oil (MR-200) and a small glass ball, which were selective to high ROS levels. The THA also demonstrated changes in electrical resistance (decrease in Δ*R*: ~ 14.60 kΩ), and stretchability (~ 127% increase in breaking strain), which may be used for screening-based applications. The THA demonstrated remarkable biocompatibility with Caco-2 (colon cancer) cells, alongside ROS scavenging and downregulation of *TNF-α* and *IL-1β*. Hence, this platform provides a viable pathway for fabricating bionic and biomimetic actuators for viscous liquid and solid transport.

**Supplementary Information:**

The online version contains supplementary material available at 10.1007/s40820-026-02257-x.

## Highlights

- Taking inspiration from the peristaltic motion of gastrointestinal system, an reactive oxygen species (ROS)- and near infrared (NIR)-responsive conductive thermo-hydrogel actuator (THA) was fabricated for viscous fluid and solid transport.
- Incorporation of PD@PDA nanoparticles provided ROS- and NIR-specific actuation properties to the THA which was confirmed by the directional movement of silicon oil (MR-200) and a small glass ball.
- Finally, the THA demonstrated ROS scavenging and downregulation of TNF-α and IL-1β, thereby highlighting its effectiveness as a bionic and biomimetic platform.

**Supplementary Information:**

The online version contains supplementary material available at 10.1007/s40820-026-02257-x.

## Graphical abstract

**Figure:**
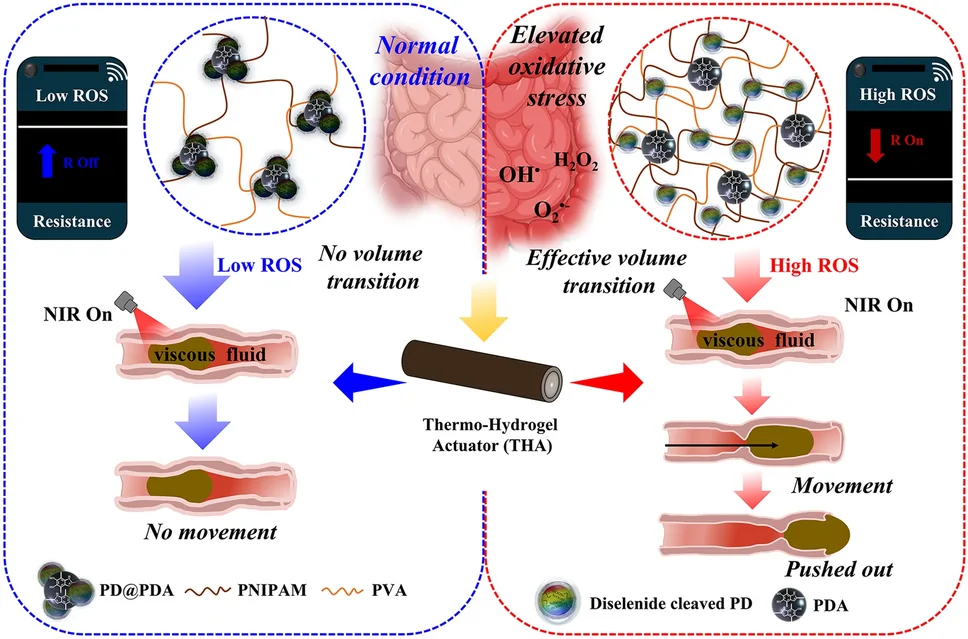

## Introduction

Soft actuators represent a promising research area owing to their capacity to transform energy from external stimuli, such as light and magnetic or electric fields, into mechanical energy [1–4]. Therefore, these systems are developed for replicating complex biomimetic motion of muscles and other organs for bionic and biomedical applications, including drug delivery, solid and fluid transport, prosthetics, and soft robotics [5–8]. An example of such a biomechanical motion is peristalsis, the process by which the movement of fluid and food materials is transported through the gastrointestinal tract, particularly the large and small intestines and the colon [9, 10]. However, in the case of redox imbalance resulting in elevated levels of ROS and inflammation, the enteric nervous system is affected, paralyzing the surrounding muscles and causing aperistalsis and restriction of flow in the gastrointestinal tract [11–13]. Several studies have attempted to fabricated actuating systems that mimic the peristaltic movement of living organism like small arthropods, adult sperm whales, and various tubular organs. However, they are mostly based off exogenous stimuli, composed of materials like inorganic composite materials, toxic conductive fillers, and involve complicated fabrication procedures [14–22]. To date, soft actuators capable of exhibiting intestinal peristaltic motion in response to endogenous stimuli, such as high levels of reactive oxygen species (ROS), remain unreported, potentially constraining future bionic applications [23, 24]. Therefore, actuators designed to mimic gastrointestinal motility require ROS responsiveness to maintain proper actuating motion, even under conditions of redox imbalance.

Thermoresponsive hydrogels are frequently employed in soft actuators owing to their volume-transitioning ability, which facilitates the alteration of the pore size and mechanical properties, driving shrinking and expansion-based actuation performance [25–27]. Furthermore, combining these thermo-hydrogel actuators with other external stimuli, such as pH, electric field, magnetic force, and light, by incorporating various nanofillers can extend their scope of application as dynamic and adaptive systems [28–30]. Such thermo-hydrogel-based actuating platforms have been designed by incorporating various technicalities in their designs such as helices, self-oscillating deformations, displacement amplification, alongside having response toward NIR. However, their applicability in selectively biomimicking gastrointestinal motion under oxidative stress and disease specific response toward NIR is hindered by their lack of the necessary endogenous-stimuli-responsive behavior, and their lack of practical working conditions aimed toward targeted drug screening and liquid/solid mass transport limiting their biological applicability [31–35]. Furthermore, these hydrogel-based actuation platforms lack electroconductive properties with respect to endogenous stimuli, which may help to correlate with the presence or absence of elevated oxidative stress in an in vitro model for screening-based applications. To overcome these challenges, thermo-hydrogel actuators may be combined with oxidative stress-responsive nanoparticles that possess electroconductivity and selective photothermal behavior to achieve specific actuation and screening functions [36, 37]. Moreover, their oxidative stress-responsive structures may enable the necessary distinguished actuation to mimic bionic functions in gastrointestinal motility-based actuation systems.

Herein an ROS- and NIR-responsive tubular conductive THA was fabricated for biomimicking intestinal peristaltic motion. The actuating system was fabricated by incorporating ROS-responsive diselenide polymer dots (PDs) loaded onto polydopamine (PDA) nanoparticles (PD@PDA) in a poly(*N*-isopropylacrylamide) and polyvinyl alcohol (PNIPAM/PVA) matrix. In addition to this, with the presence of elevated ROS, PDA is released, providing selective photothermal heat generation owing to a change in the structure of PDs. In addition, the LCST behavior of PNIPAM in combination with selective NIR-responsiveness promotes differential actuation behavior under oxidative stress imbalance. Experiments demonstrating the movement of viscous silicone oil via colon-mimicking peristaltic motion showed the selective actuation behavior of the THA. The presence of PD@PDA in a PNIPAM/PVA matrix ensures targeted and selective generation of peristaltic motion, tunable conductivity and fluorescence in addition to being biocompatible for future screening-based applications. Furthermore, the THA ensures a facile fabrication process devoid of any complications in its design and the demonstration of its actuating and screening behavior strictly aimed toward future screening functions. Finally, the ROS- and NIR-responsive actuator effectively scavenged elevated levels of oxidative stress in the presence of colon cancer cells (Caco-2), revealing its potential as a selective actuation platform for liquid and solid transport with integrated screening capabilities.

## Experimental Section

### Materials

Alginic acid sodium salt (155,000 g mol^−1^), *N*-hydroxysuccinimide (NHS), ethyl carbodiimide hydrochloride (EDC), dopamine hydrochloride, 2-bromoethylamine hydrobromide, polyvinyl alcohol (PVA, Mw = 125,000 Da), and hydrogen peroxide (H_2_O_2_) were purchased from Sigma-Aldrich (South Korea). Fetal bovine serum (FBS) and penicillin–streptomycin solutions were purchased from Thermo Fisher Scientific Korea Ltd. (Seoul, South Korea). Phosphate-buffered saline (PBS), 0.05% (w/v) trypsin-ethylenediaminetetraacetic acid (trypsin–EDTA) 1 × solution, and Dulbecco’s modified Eagle’s medium (DMEM) were procured from Gibco BRL (Carlsbad, USA). Calcein-acetoxymethyl (Calcein-AM), propidium iodide (PI), dihydroethidium (DHE), and DCFH-DA were purchased from Invitrogen (Carlsbad, CA, USA). The CCD-18 co (normal human colon), Caco-2, and SNU-C2A (human colon adenocarcinoma) cell lines were used in this study to compare the normal physiological ROS condition with the elevated ROS condition observed in the case of loss of redox balance. All the cell lines were provided by the Korean Cell Line Bank (Seoul, Korea). CCD-18 co, RRID: CVCL_2379 (normal human colon cells) was purchased on September 11, 2023, along with Caco-2, RRID: CVCL_0025 (human colon adenocarcinoma cells), whereas SNU-C2A, RRID: CVCL_1709 (human colorectal adenocarcinoma cells) cancer cell lines were purchased on December 26, 2022. Notably, the cell lines were free of contaminants. CCD-18 co cells were cultured in modified Eagle’s medium (MEM; Gibco-Invitrogen, Grand Island, NY, USA) supplemented with 10% fetal bovine serum (FBS) and 1% penicillin–streptomycin solution (Thermo Fisher Scientific Korea Ltd., Seoul, Korea). SNU-C2A cells were cultured in Roswell Park Memorial Institute (RPMI) 1640 medium (Gibco-Invitrogen, Grand Island, NY, USA) supplemented with 10% FBS and 1% penicillin–streptomycin solution. Caco-2 cells were cultured in MEM supplemented with 20% FBS and 1% penicillin–streptomycin solution. The cultures were maintained at 37 °C with 5% CO_2_, with the medium refreshed bi-daily. Upon reaching 80%–90% confluence, the cells were detached using 0.05% w/v trypsin–EDTA 1 × and centrifuged to obtain cell pellets and the resulting cell suspension for in vitro experiments. Na_2_Se_2_ stock solutions, diselenide-crosslinked dopamine-conjugated alginic acid polymer (Alg-Dp-diSe) and PD, and PDA was synthesized according to previously published reports [38, 39]. Similarly, the ROS- and NIR-responsive nanoparticle PD@PDA was loaded in a 10:1 ratio, and the process was followed according to a previous report [40].

### Fabrication of ROS- and NIR-Responsive THA

An acrylonitrile butadiene styrene (ABS) tube with a length of 45 mm and inner diameter of 2.5 mm was used as the template; an ABS rod with a diameter of 1.5 mm was placed concentrically within the ABS tube of length 60 mm. Subsequently, the prepolymer solution was prepared. Typically, NIPAM (250 mg), PD@PDA (50 mg), BIS (20 mg), and PVA (80 mg) were added to 5 mL deionized water, which was treated with an ultrasonic homogenizer for 10–15 min to obtain a homogeneous solution. Subsequently, APS (20 mg) and TEMED (10 µL) were added to the prepared solution. The final solution was then injected into the self-made device (as mentioned above) and left for 12 h. The whole process, from the preparation of the prepolymer solution to the insertion into the mold, was carried out at 0–5 ºC. After complete hydrogelation, the obtained hydrogel was separated from the mold and washed with deionized water (DIW).

### H_2_O_2_ Treatment of ROS- and NIR-responsive THA to Analyze the Effects of its Interaction with Simulated Elevated Oxidative Stress Conditions

To simulate the elevated oxidative stress-based microenvironment, the THA (inner diameter: 1.5 mm, outer diameter: 3.2 mm, wall thickness: 0.6 mm, and length: 2 mm) was treated with 100 μL of 0.1 mM H_2_O_2_ for 3 h at 37 ºC in a shaking incubator. The THA was then washed with 50 µL PBS to remove excess H_2_O_2_ solution which was followed by further analysis.

### Electrical Resistance, Wireless Response, and Optical Property Analysis of ROS- and NIR-Responsive THA

The electrical resistance of the THA samples was assessed using a Keithley 2450 sourcemeter (at room temperature). The following measurement parameters were applied to the sourcemeter: compliance: 1 A, 1 V for the BIAS level, DC BIAS function type with a 0.01-s measure delay, using a 2-electrode DC system (*n* = 3 for each sample). For the NIR on/off study, the THA samples after treatment with 0–0.1 mM H_2_O_2_ for 3 h at 37 ºC were irradiated with 808 nm NIR light for 2 min at 1.5 W cm^−2^ power. An electronic wireless system was constructed by connecting a self-prepared wireless device to the hydrogel and transmitting the results to a smartphone via a Bluetooth connection. The prepared system consisted of a self-made electronic circuit, microcontroller (Arduino Uno), and Bluetooth module (AppGosu) [41]. The NIR on/off study was similar to that described for the sourcemeter analysis. For the studies of FL intensity signals, THA samples of size 10 mm treated with 0–0.1 mM were cut into small slices of dimensions 2 × 2 × 2 mm^3^. The THA samples were then placed in a confocal dish and observed under CLSM microscope at magnification: 20x. The THA samples of size 2 mm (inner diameter: 1.5 mm, outer diameter: 3.2 mm, and wall thickness: 0.6 mm) were treated with 0–0.1 mM H_2_O_2_, followed by observing under the fluorescence (FL) microscope at 2.5 × 1000 µm magnification. All experiments were performed for *n* = 3 samples. For the NIR on/off study, the NIR treatment was carried out in a manner similar to the process mentioned for the electrochemical analyses.

### Thermoresponsive Viscoelastic Property Analysis of ROS- and NIR-Responsive THA

The thermoresponsive viscoelastic properties of THA were analyzed using a rheometer (Haake Mars, Thermo Fisher, Germany) with parallel plate of 40 mm thickness, separated by a gap of 0.5 mm. Temperature-dependent measurements were conducted with a heating rate of 1 °C min^−1^, frequency set to 1 Hz, and steady-state measurements at 10 Pa. Temperature sweep was performed at 10–80 °C, with a constant frequency of 1 Hz and strain of 1% (*n* = 3 per sample). The reversible oscillatory temperature sweep was assessed over four heating and cooling cycles between 25 and 37 °C (± 1 °C min^−1^).

### Investigation of Actuation and Biomimicking of Intestinal Peristalsis for Transport of Viscous Liquid and Solids by the ROS- and NIR-Responsive THA

The investigation of actuation performance was carried out by treating the THA with 0–0.1 mM H_2_O_2_ for 3 h at 37 °C, which was followed by subjecting the THA to NIR irradiation for 2 min at 1.5 W cm^−2^ power for a maximum of 2 min to analyze the variations in bending and diameter. Furthermore, the THA was injected with MR-200 silicone oil, which had been previously mixed with rhodamine red dye. The THA had the following dimensions: length, 40 mm; inner diameter: 1.5 mm, outer diameter: 3.2 mm, and wall thickness: 0.6 mm. Then, the THA was irradiated with 808 nm NIR light to push the silicone oil from one end of the vessel to the other end, and the time taken was noted. A similar process was followed for analyzing the actuation performance of the movement of a solid glass ball across the THA. All experiments were repeated for *n* = 3 samples.

### In Vitro Cytotoxicity Assessment, Rheological, and Electrochemical Property of the ROS- and NIR-responsive THA

The in vitro cytotoxicity of THA was analyzed using CCD-18 co as normal cells and Caco-2 and SNU-C2A as colorectal adenocarcinoma cells. First, the cell suspension with a cell density 10^5^ cells 50 µL^−1^ was prepared. Then, the THA (length: 5 mm, inner diameter: 1.5 mm, outer diameter: 3.2 mm, and wall thickness: 0.6 mm) was put onto a confocal dish and incubated with cell suspension (50 µL) for a time period of 12 h at 37 °C in humidified 5% CO_2_. After incubation, the hydrogel was washed with PBS (50 µL). The cells were then stained and incubated with calcein AM (150 μL) for 30 min followed by propidium iodide (PI) (50 μL) for 5 min. The stained hydrogel was observed under CLSM at a magnification of 100 μm to observe live and dead cells. The in vitro rheological properties of THA were analyzed by treating the hydrogel with Caco-2 and SNU-C2A cancer cell lines and CCD-18 co cells as the normal cell line. The cell treatment was conducted by preparing a cell suspension of density 10^5^ cells 100 µL^−1^, following which the suspension (100 µL) was transferred onto the THA (length: 5 mm, inner diameter: 1.5 mm, outer diameter: 3.2 mm, and wall thickness: 0.6 mm) in 24-well cell culture plates. Consecutively, the THA hydrogel was incubated in an incubator for 12 h at 37 °C and humidified 5% CO_2_. After incubation, the remaining cell suspension was removed and the hydrogel actuator was washed with PBS. The conditions employed for the in vitro rheological experiments was identical to those for the elevated levels of ROS (*n* = 3 samples). In addition, for the in vitro electrochemical analysis, the THA (length: 5 mm, inner diameter: 1.5 mm, outer diameter: 3.2 mm, and wall thickness: 0.6 mm) was treated with 10^5^ cells 100 µL^−1^ and incubated for 12 h at 37 ºC in humidified 5% CO_2_. After incubation, the remaining cell suspension was removed and the hydrogel was washed with PBS. Identical conditions were employed for the in vitro electrochemical experiments and the cancer-simulated conditions (*n* = 3 samples).

## Results and Discussion

### Fabrication and Working Mechanism of the ROS- and NIR-responsive THA for Intestinal Peristalsis Motion

**Fig. 1:**
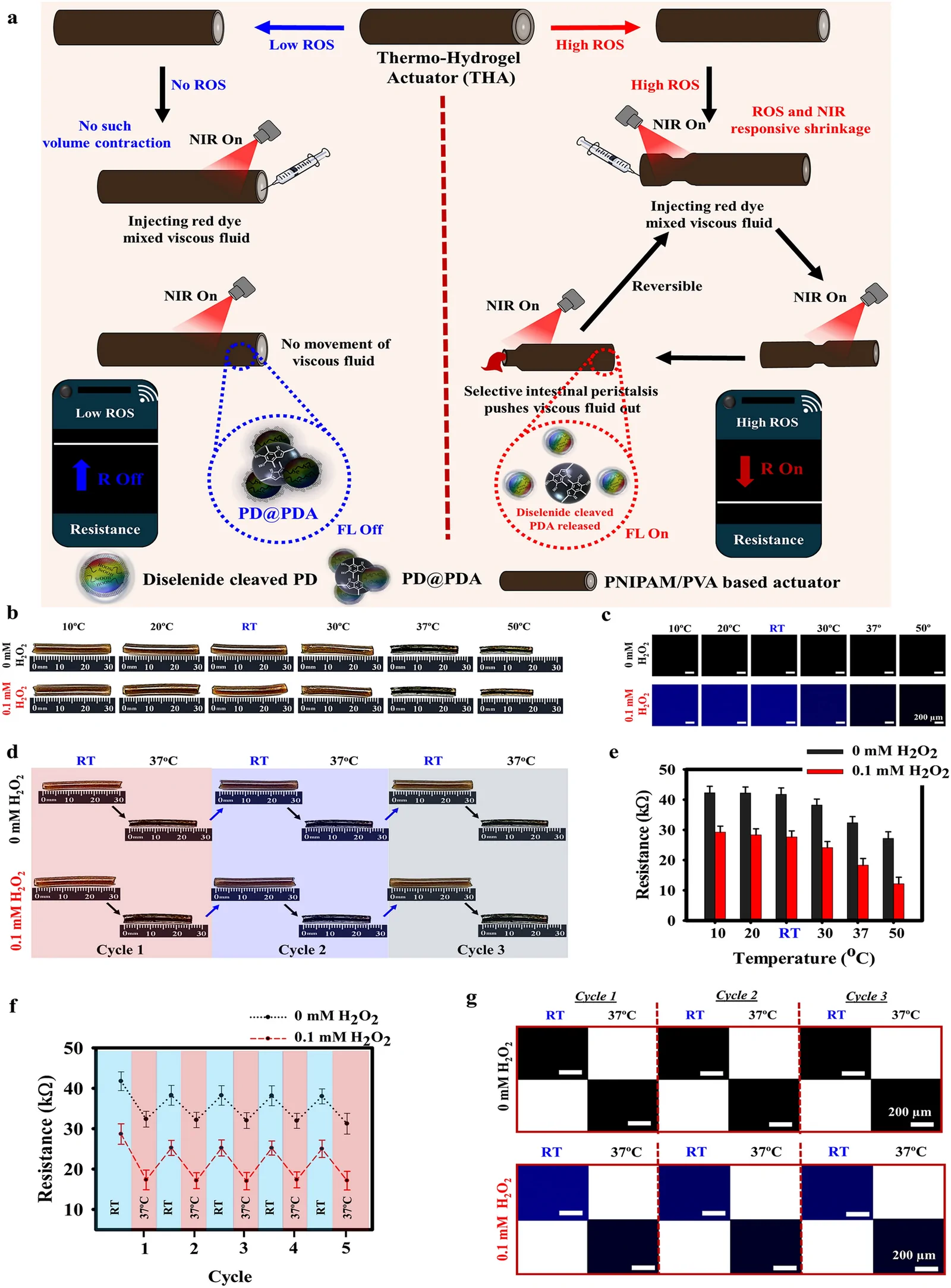
Fabrication and working mechanism of the ROS- and NIR-responsive THA for intestinal peristalsis motion: **a** Schematic illustration of the working mechanism. **b** Images of thermoresponsive change in length of the THA after exposure to 0–0.1 mM H_2_O_2_. **c** Variations in CLSM optical FL images after exposure to various temperature before and after treatment with 0–0.1 mM H_2_O_2_. **d** Reversibility images of THA after keeping at room temperature (25 °C) and body temperature (37 °C) following treatment with 0–0.1 mM H_2_O_2_ for 3 cycles. **e** Trends in the sourcemeter resistance signals of the THA after exposure to various temperature before and after treatment with 0–0.1 mM H_2_O_2_. **f** Resistance reversibility at RT and 37 °C in presence of 0–0.1 mM H_2_O_2_. **g** Fluorescence signal reversibility via optical CLSM images of the THA after keeping at room temperature (25 °C) and body temperature (37 °C) at RT and 37 °C in the presence of 0–0.1 mM H_2_O_2_ for 3 cycles

An ROS- and NIR-responsive THA was fabricated to mimic gastrointestinal peristalsis motion for transport of viscous liquids, solids, and screening functions. The THA was fabricated by incorporating PD@PDA nanoparticles into a PNIPAM/PVA matrix. The presence of high levels of ROS during oxidative stress imbalance causes the release of PDA from PD@PDA resulting in the selective photothermal heat generation and the successive differential actuation of THA. Additionally, the release of PDA affects the fluorescence and resistance signals which helps to correlate with ROS levels in the presence of Caco-2 cancer cells. Finally, the presence of PD@PDA in THA provides the ROS-scavenging and anti-inflammatory activities toward cancer cells, demonstrating its anti-oxidative properties (Fig. 1a).

Briefly, ROS responsive PDs were fabricated by the hydrothermal carbonization of a diselenide-crosslinked alginate polymer at 60 °C for 24 h. The ^1^H-NMR spectra of the PD was analyzed at 400 MHz. The results showed the presence of the alginate peaks at δ 3.55–4.36 occurring as a multiplet as in Fig. S1. Moreover, aromatic peaks of the catechol moiety were observed at δ 6.5–7.0 and the NH_2_–CH_2_(c)–CH_2_(d)–Ar peaks appeared at δ 2.77–3.05. The diselenide crosslinking was shown by the presence of peaks at δ 1.78–3.31 for NH_2_–CH_2_(a)–CH_2_(b)–Se. Then, the ROS- and NIR-responsive nanoparticles were fabricated by physically loading PD with PDA in a 10:1 weight ratio to obtain PD@PDA according to a previously published procedure. This was followed by the fabrication of the tubular conductive THA by incorporating PD@PDA into a PNIPAM/PVA matrix followed by polymerization with *N,N′*-methylenebisacrylamide (BIS) and ammonium persulfate (APS). Following treatment of the hydrogel with 0–0.1 mM H_2_O_2_, the hydrogel was subjected to various temperature (10, 20, RT, 30, 37, and 50 ºC). The thermoresponsive behavior of the THA is shown in Figs. 1b and S2a, where both the 0–0.1 mM H_2_O_2_-treated THA demonstrated decrease in length. Then, the effect of ROS on the temperature responsive fluorescence signals of the THA was analyzed using CLSM images. For the 0 mM H_2_O_2_-treated THA sample at all temperatures (10, 20, RT, 30, 37, and 50 ºC) did not show any fluorescence signals as shown in Fig. 1c. This is owing to the presence of PDA and diselenide bonds in the PD@PDA nanoparticles in the THA. The diselenide bonds produce quenching effect via aggregation caused quenching (ACQ), whereas PDA contributes to the quenching effect by means of the FRET effect with PDA acting as the acceptor and PD acting as the donor [42–48]. However, upon treatment with 0.1 mM H_2_O_2_, the FL signals are recovered as seen from the CLSM images which were owing to the presence of a graphitic core of the PDs. Additionally, when the THA was subjected to various temperature, the FL signals demonstrated a decrease in FL intensity at higher temperature. This was caused by the ACQ effect of PNIPAM caused by the volume contraction and subsequent aggregation of the PNIPAM polymer chains at temperature higher than its LCST resulting in the close packing of the cleaved PD@PDA nanoparticles. Additionally, the reversibility of the thermoresponsive behavior of THA is shown in Figs. 1d and S2b, with the THA demonstrating reversible decrease in dimension between RT and 37 ºC for 3 cycles in presence of both 0–0.1 mM H_2_O_2_. The influence of ROS and temperature on the sourcemeter resistance of the THA was analyzed after treatment with 0–0.1 mM H_2_O_2_. The results showed the decrease in resistance for 0 mM H_2_O_2_-treated THA owing to the decrease in THA dimension caused by the volume transition at LCST of PNIPAM as given in Fig. 1e. However, the resistance of the THA treated with 0.1 mM H_2_O_2_ demonstrated further decrease which was caused by the effect of ROS on the incorporated PD@PDA containing diselenide bonds that undergo cleavage resulting in the release of PDA that increased the electronic mobility and area of contact between the particles in the matrix. Similarly, the resistance reversibility study of the THA showed excellent thermoreversible behavior after dipping in water in presence of both 0–0.1 mM H_2_O_2_ as shown in Fig. 1f. Additionally, it was observed that the resistance value at 37 °C and 0.1 mM H_2_O_2_ treatment shows a decrease which is owing to the diselenide cleavage followed by the release of PDA in combination with the LCST-based volume transition of the PNIPAM in the matrix. The reversibility analysis is performed by dipping the THA sample in 500 µL DDW for a period of 2 min in order for the THA to reswell and obtain its previous dimension. During this process, following the 1st cycle at RT and 37 °C, the sample undergoes rapid reswelling at the start of the 2nd cycle at RT lowering the resistance value. However, after successive cycles of shrinking and reswelling, the swelling rate reaches an equilibrium which stabilizes the resistance value, thereby accounting for the lowered resistance at RT from the 2nd to 5th cycle as compared to the 1st cycle. Moreover, as in Fig. 1g, the THA demonstrated thermoresponsive reversibility of FL signals at RT and 37 ºC highlighting its temperature responsive behavior.

The LCST behavior of the THA was analyzed using temperature sweep at constant sheer via rheology. The LCST of the THA for 0 mM H_2_O_2_ was observed to be ~ 29 °C as compared to 0.1 mM H_2_O_2_ which showed ~ 32 °C as shown in Fig. S3a. This shift in the LCST was owing to the diselenide cleavage and the release of PDA which induced hydrogen bonding in the matrix and hydrophilicity shifting the LCST to a higher temperature. This shift in the LCST and various phases of the THA was further visualized using the Tan δ plot as given in Fig. S3b. The LCST behavior of THA and the influence of the diselenide cleavage followed by PDA release on its shift were verified using the dynamic scanning calorimetry (DSC) analysis. The results showed the presence of the LCST peak of THA at ~ 29 °C which shifted to ~ 32 °C following treatment with 0.1 mM H_2_O_2_ as shown in Fig. S4. Additionally, the presence of the melting peaks (*T*_*m*_) was observed at ~ 105 °C which also demonstrated a shift to ~ 113 °C. This was owing to the diselenide cleavage and PDA release which influenced the shift of H-bonding, thereby providing stability to the matrix [49–52]. Moreover, the LCST and its shift were also confirmed by the thermomechanical analyzer (TMA). The results given in Fig. S5 show the presence of LCST of the THA actuator after 0 mM H_2_O_2_ treatment at ~ 29 °C, with the THA showing contraction behavior from the change in initial dimension. Furthermore, the LCST shifted to ~ 32 °C which was in agreement with the temperature sweep at constant sheer and DSC analysis [53]. This was followed by analyzing the effect of elevated levels of ROS on the thermoresponsive viscoelastic property of the THA. The temperature sweep of the THA at a constant frequency of 1 Hz and constant strain of 1% after treatment with 0–0.1 mM H_2_O_2_ showed that at 0 mM H_2_O_2_ the THA underwent structural collapse and loss of elasticity at ~ 64 °C when compared to the 0.1 mM H_2_O_2_-treated THA (~ 71 °C), as in Fig. S6. The shift in the collapse temperature of the THA polymer network (G” > G’) demonstrates the influence of H-bonding after cleavage of diselenide bonds releasing PDA into the matrix. Additionally, the cleavage of diselenide bonds leading to the release of PDA and the successive influence of H-bonding in the THA matrix was confirmed suing the FT-IR analysis. The 0.1 mM H_2_O_2_-treated THA showed shifts in the following stretching frequency from 3319 to 3241 cm^−1^ (–OH), 2996–2905 cm^−1^ (secondary amide), 1652–1601 cm^−1^ (–NH–C=O, amide I band), and 1549–1497 cm^−1^ (–NH–C=O, amide II band) when compared to the 0 mM H_2_O_2_-treated sample as shown in Fig. S7. This shift in the H-bonding influences the physical and the mechanical property of the actuator. The thermogravimetric analysis of the THA demonstrated degradation of the matrix in various steps after treatment with the structural water loss observed at 0–121 °C. This was followed by the degradation of the PD and PDA at with a shift observed for the 0.1 mM H_2_O_2_-treated THA showing the increase in stability. The PNIPAM and alginate-based PD in the matrix degraded at 330–477 °C and finally, the last step involved the degradation of PVA from 478–600 °C as shown in Fig. S8. Furthermore, owing to the presence of the harsh acidic microenvironment of the overall gastrointestinal system, the THA was subjected to stability and selectivity analysis. The results in Fig. S9a, b show the good qualitative and quantitative stability of the actuator at various pH solutions for a period of up to 5 days. Additionally, the specificity of the THA toward ROS was analyzed by measuring the resistance signals of the THA subjected to various pH solutions. The results in Fig. S9c showed that the THA did not undergo any change in its electronic resistance signals which further helped to verify its ROS-specific behavior. From the above demonstrations, it is evident that the diselenide-crosslinked PD@PDA in the presence of high levels of ROS undergoes a redox reaction which causes the dissolution of the diselenide bonds into seleninic acid (-SeOOH) or in certain instances selenenic acid (-SeOH) [54–58]. This consequently triggers the release of PDA affecting various properties of the THA and causing tunable shifts in the ROS and thermoresponsive properties of the actuator.

### ROS- and NIR-Responsive Behavior and Actuation of the THA

**Fig. 2:**
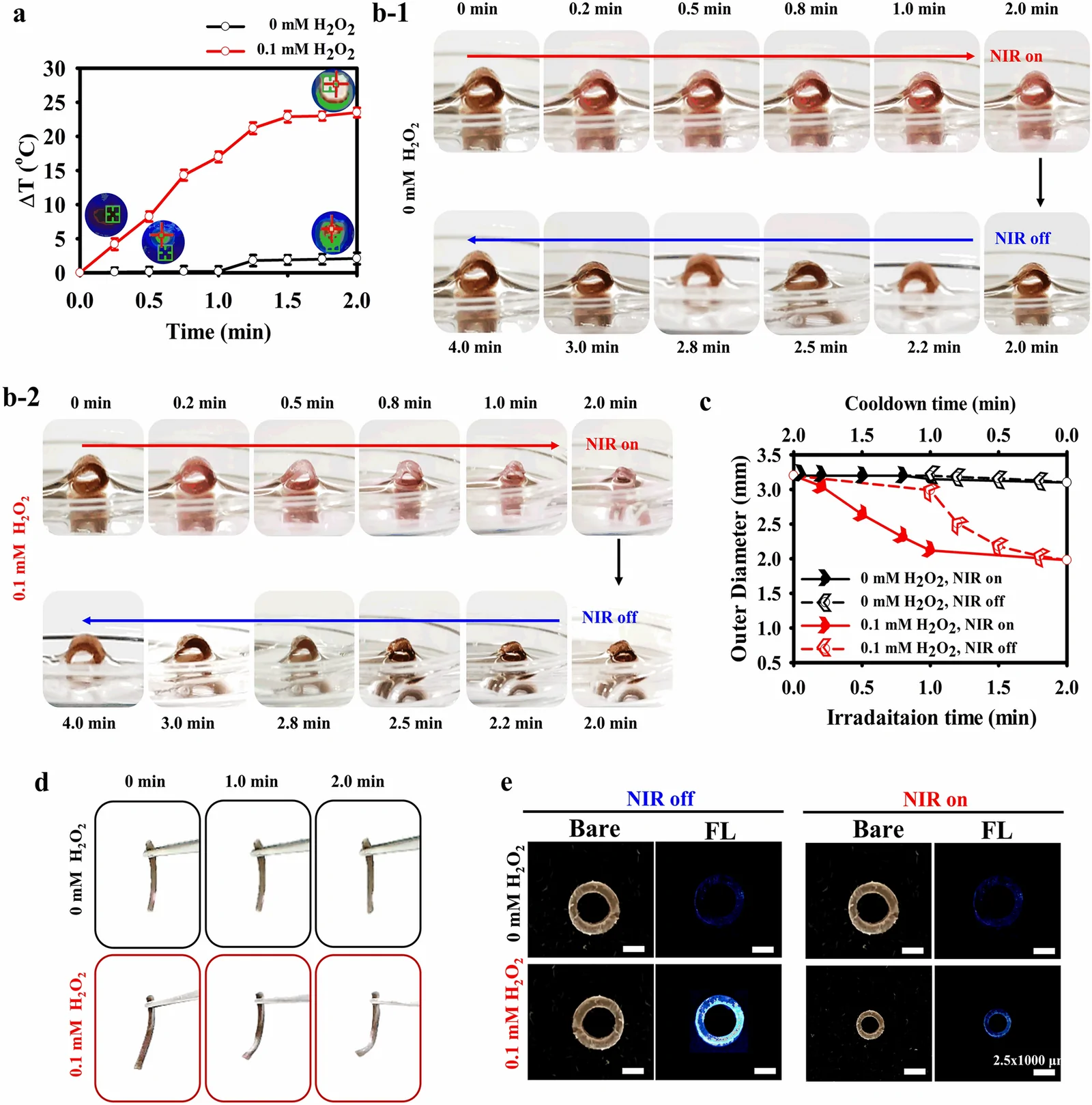
ROS- and NIR-responsive behavior and actuation of the THA: **a** Photothermal heat generation in presence of 0–0.1 mM H_2_O_2_ with NIR images after dipping in water at 1.5 W NIR laser power. Images of the THA diameter change and reversibility after dipping in water in presence of NIR following treatment with 0 (**b-1**) and 0.1 mM H_2_O_2_ (**b-2**). **c** Change in diameter and reversibility of the THA with NIR irradiation and cooldown with dipping in water. **d** Actuation of the THA via bending in presence of laser. **e** Fluorescence microscopy images before and after NIR of the THA following treatment with 0–0.1 mM H_2_O_2_

Following the fabrication, and analysis of its working mechanism, the tubular conductive THA was then studied for its ROS- and NIR-dependent properties. From the results given in Fig. S10a, it can be seen that the THA demonstrated selective photothermal heat generation without dipping in water only in case of 0.1 mM H_2_O_2_-treated hydrogel as compared to 0 mM H_2_O_2_-treated THA. Additionally, the photothermal heat generating capacity of the THA increased with increasing laser power. This was followed by assessing the photothermal effect after dipping the THA to observe its photothermal heat generating efficiency without loss of water. It can be seen that compared to without water the heat generation efficiency decreases in presence of water (Fig. S10b). Therefore, at the end of the analyses, keeping in mind the harmful effects of laser power for biomedical applications, 1.5 W cm^−2^ was chosen as the optimum power source. In presence of 1.5 W cm^−2^ power, following treatment of THA with 0–0.1 mM H_2_O_2_ the photothermal heat generation demonstrated selective temperature elevation for the 0.1 mM H_2_O_2_-treated THA, as given in Fig. 2a. As the THA was introduced to high levels of ROS the incorporated PD@PDA nanoparticles underwent cleavage of the diselenide bonds owing to the redox reaction with H_2_O_2_ and triggered the release of PDA. This formed the foundation of its ROS and NIR-selective actuation properties and was followed by analyzing the change in the diameter of the THA under NIR light after treatment with 0 and 0.1 mM H_2_O_2_. From Fig. S11, it can be seen that the outer diameter of the hydrogel decreases in presence of 0.1 mM H_2_O_2_ owing to the released PDA which generates photothermal heat that affects the volume transition of the PNIPAM/PVA matrix [59–62]. However, the change in diameter is an irreversible process as the water pumped out during the process cannot be completely reabsorbed due to evaporation. In comparison, the tubular conductive THA when dipped in water as shown in Fig. 2b-1 and 2b-2, demonstrates complete reversible behavior of its changing outer diameter, further establishing its potential for repeated actuation. This phenomenon of reversible behavior of the THA diameter after dipping in water was confirmed from the quantitative studies in Fig. 2c. This was followed by analyzing the bending performance of the THA under NIR light. Figure 2d shows that the THA treated with 0 mM H_2_O_2_ did not show any appreciable bending in comparison to the 0.1 mM H_2_O_2-_treated THA. The reason for the selective actuation in case of elevated ROS conditions is owing to the cleavage of diselenide bonds in the PD@PDA nanoparticle leading to the release of PDA that provides selective photothermal heat generation and successive bending-based actuation of the THA. Finally, the fluorescence microscopy images of the THA after treatment with 0 and 0.1 mM H_2_O_2_ was analyzed to observe the NIR-responsive thermoresponsive effect on the FL signal. From Fig. 2e, it can be seen that for 0 mM H_2_O_2_, the THA does not show any FL signal with NIR off, however, upon treatment with 0.1 mM H_2_O_2_, the THA demonstrated regain in FL signal which was quenched with NIR owing to the aggregation in the PNIPAM/PVA matrix induced by the LCST behavior-based volume transition of THA.

### Redox and NIR Responsive Changes to the Optical and Electrical Signals of the THA

**Fig. 3:**
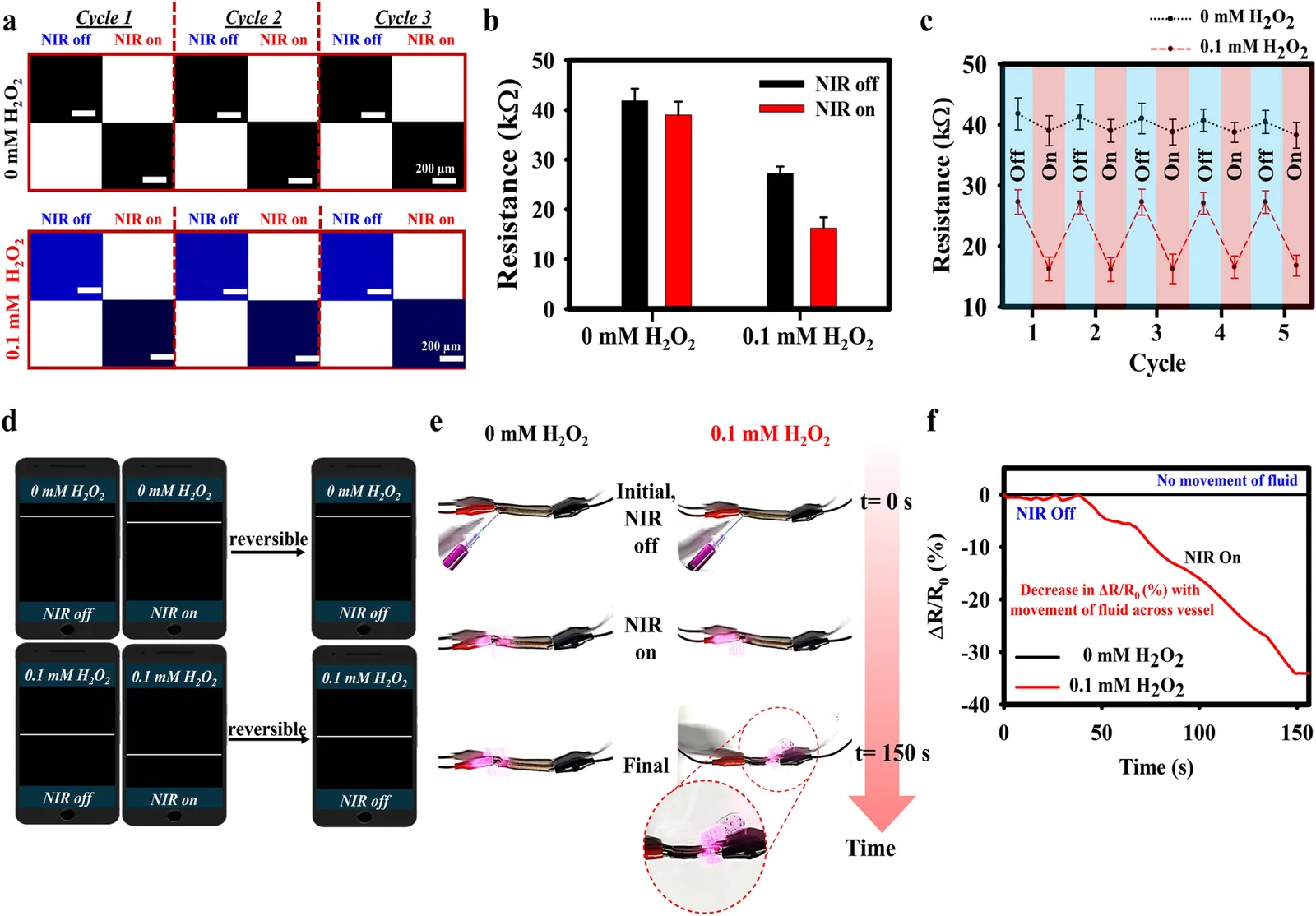
Redox and NIR responsive changes to the optical and electrical signals of the THA: **a** FL intensity reversibility analysis in presence and absence of NIR for 3 cycles following treatment with 0–0.1 mM H_2_O_2_. **b** Influence of ROS and NIR on the sourcemeter resistance signal of the THA after dipping in water. **c** Reversibility of the obtained resistance signal of the THA in the presence and absence of NIR after dipping in water for 5 cycles. **d** Wireless resistance signals of the THA and the influence of 0–0.1 mM H_2_O_2_ alongside NIR on the obtained response. **e** Images of the procedure of the electrochemical resistance response of the THA after silicon oil MR-200 mixed with red dye is passed through it using NIR with respect to time. **f** Electrochemical resistance response of the THA after viscous silicon oil is passed through it using NIR following treatment with 0–0.1 mM H_2_O_2_

The effect of the ROS- and NIR-responsive behavior of the tubular conductive THA was then analyzed by it redox effect on the electronic and fluorescence signals. The FL intensity of the THA was quenched primarily owing to the aggregation caused quenching (ACQ) effect of diselenide bonds and the FRET effect of PDA in presence of 0 mM H_2_O_2_, with NIR off. However, with the introduction of 0.1 mM H_2_O_2_ with NIR off, resulting in the cleavage of diselenide bonds and the simultaneous release of PDA, the quenching effect is reduced which causes recovery of fluorescence. Then, in NIR on state, after treatment with 0–0.1 mM H_2_O_2_, the FL signals remain unchanged for 0 mM H_2_O_2_, NIR on state, however for 0.1 mM H_2_O_2_, NIR on state, the THA undergoes quenching effect as given in Fig. 3a. This is owing to the volume transition assisted polymeric aggregation of PNIPAM chains in the matrix caused by it LCST and NIR-selective behavior. The procedure was repeated for 3 cycles to demonstrate the thermoreversible and ROS- and NIR-selective behavior of the THA. This was followed by the sourcemeter resistance and resistance reversibility analysis of the THA after treatment with 0–0.1 mM H_2_O_2_. As given in Fig. 3b, the resistance of the of the sample in presence of NIR can be attributed to the thermoresponsive behavior of the PNIPAM/PVA matrix which undergoes contraction in volume with expulsion of water. Furthermore, the decrease in resistance is higher in presence of 0.1 mM H_2_O_2_ (NIR off: 39.01 ± 0.26 kΩ to NIR on: 16.23 ± 0.21 kΩ) as compared to 0 mM H_2_O_2_ (NIR off: 41.85 ± 0.24 kΩ to NIR on: 27.25 ± 0.13 kΩ) owing to the release of PDA which absorbs NIR light. Additionally, owing to LCST behavior the THA (without dipping in water) loses its water content and as shown in Fig. S12 its ability to return to original dimension is lost resulting in an irreversible resistance signal over 5 cycles. However, as the THA is dipped in water it, readily reabsorbs the lost water and returns to near original state, giving rise to reversible resistance signal as shown in Fig. 3c. The wireless resistance signals were analyzed to correlate the change in resistance of the THA actuator hydrogel using sourcemeter resistance. As shown in Fig. 3d, the wireless signals showed similarities in the trend observed using sourcemeter and the reversible behavior was reciprocated as well in presence of water. Finally, as given in Fig. 3e, the images from the experiment and the change in the relative resistance response of the THA actuator upon movement of silicon oil MR-200 mixed with red dye following treatment with 0–0.1 mM H_2_O_2_ with respect to time was analyzed using an LCR meter. From Fig. 3f, it can be seen that the sensitivity of THA remained at 0 as long as NIR was off for both 0–0.1 mM H_2_O_2_ treatment, however, once NIR was turned on and as the viscous fluid moved through the vessel via volume transition of PNIPAM matrix resulting in decrease of its sensitivity. For the 0 mM H_2_O_2_-treated THA, there is no movement of viscous fluid inside the actuator owing to its ROS selective NIR-responsive actuation behavior, which resulted in no change of its electrochemical sensitivity.

### Redox Responsive Changes to Physical and Mechanical Properties of the THA

**Fig. 4:**
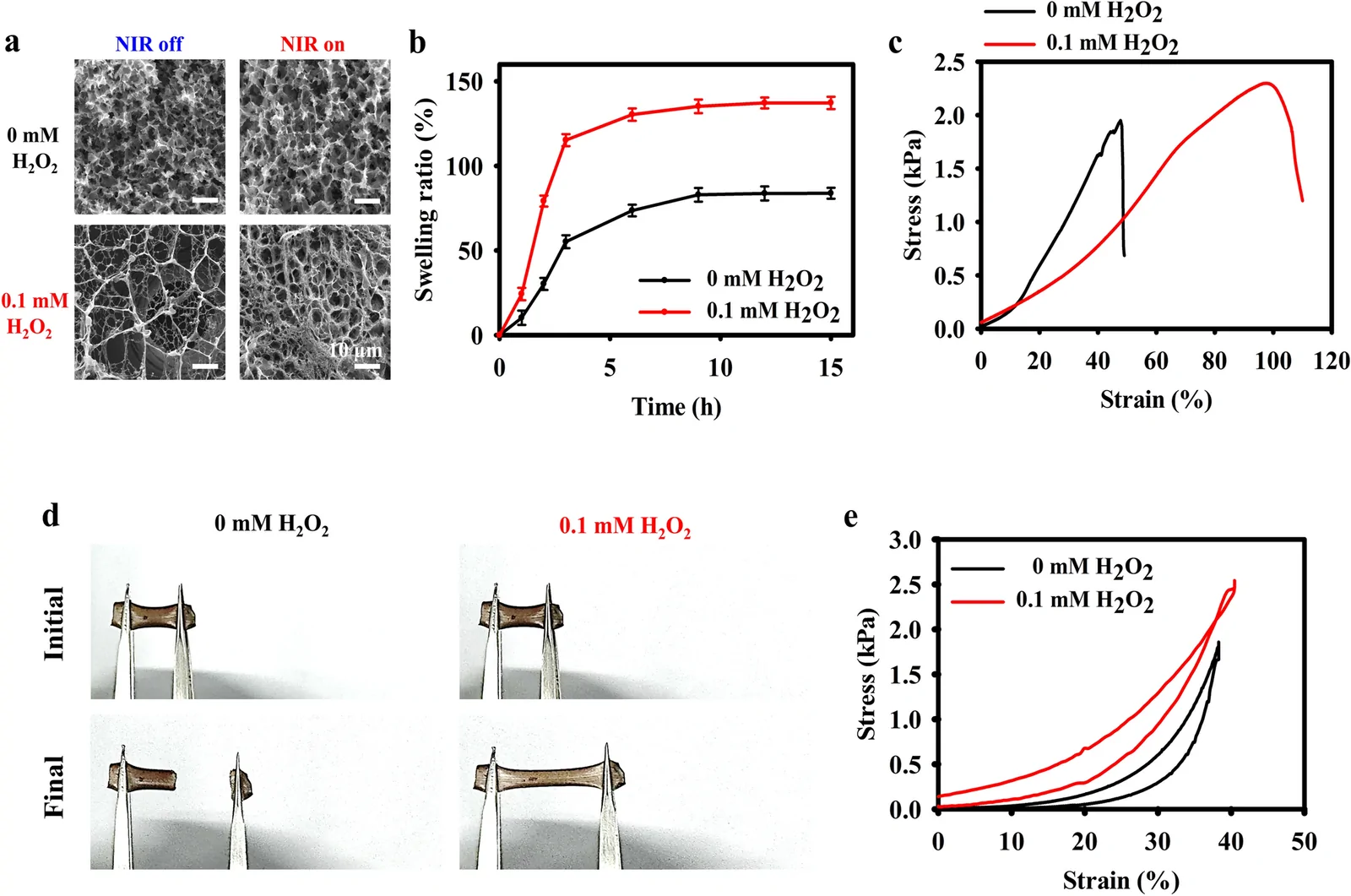
Redox responsive changes to physical and mechanical properties of the THA: **a** SEM images of variation of pore size in presence of NIR following treatment with 0–0.1 mM H_2_O_2_. **b** Swelling ratio analysis of the THA after treatment with treatment with 0–0.1 mM H_2_O_2_. **c** Trends in tensile mechanics of the THA after treatment with treatment with 0–0.1 mM H_2_O_2_. **d** Variation of stretchability shown by stretching images of the THA after treatment with 0–0.1 mM H_2_O_2_. **e** Trends in the cyclic compressive modulus of the THA after exposure to 0–0.1 mM H_2_O_2_

Following the evaluation of the redox effect of the ROS on the electrochemical and optical properties of the THA, the redox responsive changes to the mechanical and physical properties of the actuator were studied. The release of PDA following the cleavage of diselenide bonds in PD@PDA affected the water uptake and pore morphology as can be seen from Fig. 4a, wherein, the 0.1 mM H_2_O_2_-treated THA actuator hydrogel demonstrated macroporous morphology in NIR off mode as compared to 0 mM H_2_O_2_. On application of NIR irradiation the pore size of the THA actuator decreased owing to the selective photothermal effect in case of 0.1 mM H_2_O_2_-treated actuator hydrogel. Additionally, the water uptake capacity of the THA demonstrated an increase from 83.86% in case of 0 mM H_2_O_2_ treatment to 137.2% in case of 0.1 mM H_2_O_2_ as given in Fig. 4b. This is owing to the cleavage of diselenide bonds and the simultaneous release of PDA into the THA matrix that induces hydrophilic behavior. Moreover, the release of PDA in presence of 0.1 mM H_2_O_2_ affected the mechanical property of the THA, as shown in Fig. 4c. The hydrophilicity induces an increase in the elasticity of the THA as can be seen from the increase in tensile stress and breaking strain as compared to the 0 mM H_2_O_2_-treated samples. Similarly, the physical property images demonstrated the higher stretchability of the THA in presence of 0.1 mM H_2_O_2_ in comparison to 0 mM H_2_O_2_-treated samples as the stretchability showed an increase compared to the initial state as shown in Fig. 4d. Finally, the compressive modulus of the THA was analyzed following treatment with 0–0.1 mM H_2_O_2_ to observe changes in the mechanical strength. The results demonstrated the higher mechanical strength under and applied sheer for the 0.1 mM H_2_O_2_ in comparison to the 0 mM H_2_O_2_ as in Fig. 4e. This is owing to the release of PDA in the matrix from PD@PDA following the cleavage of diselenide bonds that induces an increase in H-bonding resulting in higher elasticity and mechanical strength.

### Simulation of Intestinal Peristalsis Motion by Transporting of Viscous Fluid or Solid Using the THA

**Fig. 5:**
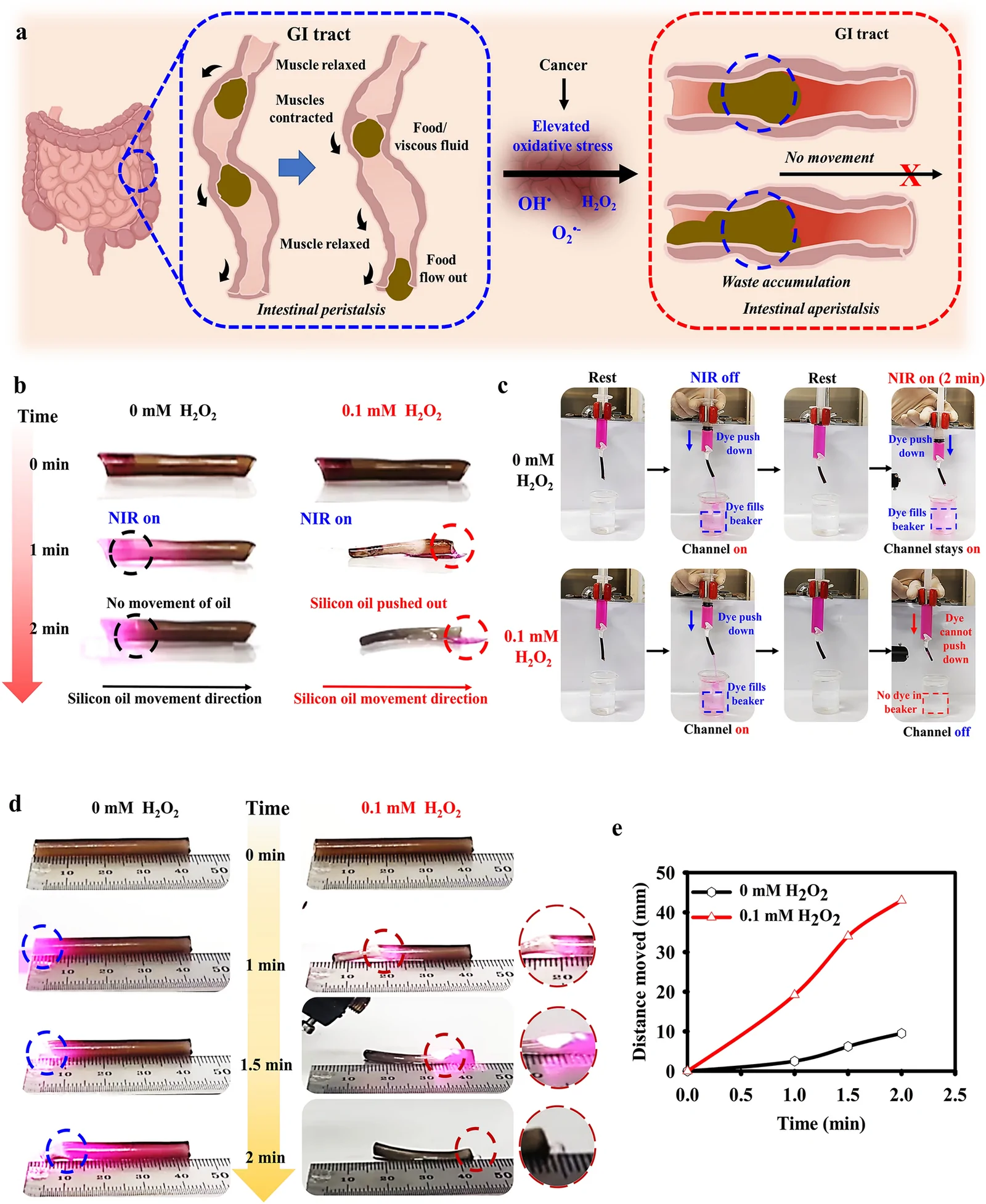
Simulation of intestinal peristalsis motion by transporting of viscous fluid or solid using the THA: **a** Schematic illustration of gastrointestinal peristalsis of solids and viscous liquids in the intestine.** b** Peristalsis of silicon oil MR-200 across the THA after exposure to 0–0.1 mM H_2_O_2_. **c** Illustration of actuation of the THA via the channel on/off analysis using silicon oil MR-200 mixed with Rhodamine dye following treatment with 0–0.1 mM H_2_O_2_. **d** Demonstration of peristalsis of solid materials through the THA like a small glass ball to mimic movement of solid or viscous food materials with time in presence of various levels of ROS. **e** Study of distance moved by the glass ball across the THA with irradiation time to show specificity toward high ROS levels

The verification and the analysis of the effect of elevated ROS on the mechanical and physical properties of the THA was followed by the analysis of its actuating properties. As food passes through the GI tract, it is moved slowly by contraction and relaxation of muscles. This particular motion is known as intestinal peristalsis and is responsible for the movement of flood and viscous fluid in the intestine. However, in case of diseases like cancer which is characterized by an increase in the levels of ROS like H_2_O_2_, the enteric nervous system is affected which paralyzes the intestinal muscle walls resulting in intestinal aperistalsis as shown in Fig. 5a. Hence, the actuation properties were designed to bio-mimic peristalsis motion for bionic functions in presence of elevated levels of ROS.

The ROS selective peristalsis motion was analyzed in the presence of MR-200 silicon oil mixed with rhodamine dye. The THA was treated with 0–0.1 mM H_2_O_2_ and then the dye mixed with oil was loaded onto one end of the THA and NIR was applied as shown in Fig. 5b. It can be seen that within 2 min the silicon oil was successfully pushed out of the THA incase of 0.1 mM H_2_O_2_-treated actuator as compared to the 0 mM H_2_O_2_. This is owing to the presence of PD@PDA in the THA that selectively undergoes cleavage of diselenide bonds and the successive release of PDA in the presence of high levels of ROS demonstrating selective NIR-responsive actuating behavior. Additionally, in another instance, the THA actuator was used to perform the channel on and off experiments by attaching to a syringe. From Fig. 5c, it can be seen that when the piston of the syringe filled with red dye is pushed with the NIR off state for the 0–0.1 mM H_2_O_2_-treated THA, the red dye inside is pushed down into the beaker as the channel remains open at 0 min of irradiation. However, afterward following 2 min if NIR irradiation, the dye is pushed down again and it can be seen that the ROS and NIR-responsive THA shrinks and transforms into off state which stops the dye from pushing out demonstrating its selective actuating behavior. Next, in another actuation experiment to simulate peristalsis, a small glass ball was put inside the THA actuator hydrogel and was pushed out with the help of NIR as given in Fig. 5d. From the images, it can be seen that the ball barely moves in the case of the 0 mM H_2_O_2_-treated THA as compared to the 0.1 mM H_2_O_2_-treated actuator. This is due to the ROS and NIR-selective behavior of the actuator which undergoes contraction by pushing water out from the polymeric hydrogel matrix. Moreover, a ruler was used to track the distance moved by the glass ball corresponding to the length of the vessel as in Fig. 5e. The results demonstrated that the 0.1 mM H_2_O_2_-treated THA was successful in moving the ball out of the vessel as compared to 0 mM H_2_O_2_-treated actuator demonstrating its selective actuating behavior. Additionally, the pressure generated during the contractile motion of the THA during its actuation process was estimated using the compressive analysis with a UTM. For the movement of a bolus from tubular constructs in the body like the gastrointestinal tract and the esophagus, the pressure generated during the contractile motion is estimated to be in the range of 0.8–33 kPa [63, 64]. From the results in Fig. S13, it can be seen that the compressive stress generated by the THA after treatment with 0.1 mM H_2_O_2_ and exposing to NIR showed a decrease owing to the ROS- and NIR-specific volume transition of the actuator in comparison to the 0 mM H_2_O_2_-treated actuator. Additionally, the compressive stress value generated by the THA following NIR irradiation falls within the range reported for bolus movement demonstrating its capability for actuating performance.

### In Vitro Study of THA in Presence Cancer Cells for Analyzing Change in Mechanical and Electrical Property

**Fig. 6:**
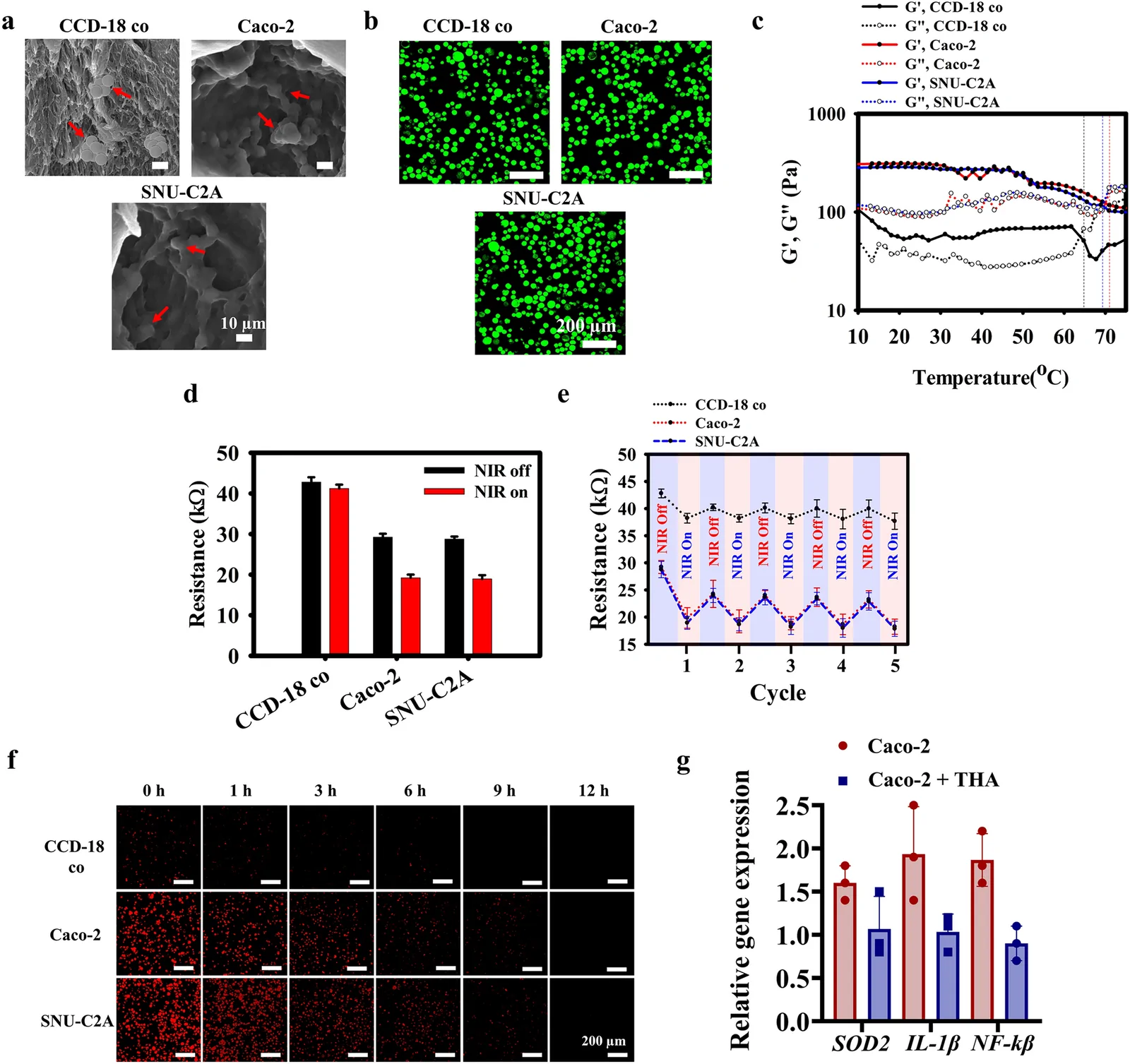
In vitro study of THA in presence cancer cells for analyzing change in mechanical, and electrical property: **a** SEM images of the THA after incubation with normal and cancer cells. **b** Live and dead assay of the normal and cancer cells incubated with THA. **c** Change in thermoresponsive viscoelastic property of the THA by the influence of normal and cancer cells. **d** Resistance change via sourcemeter analysis of the THA. **e** Resistance reversibility of the THA. **f** ROS-scavenging effect of the incorporated diselenide nanoparticles in the THA in presence of CCD-18 co (normal) cells and Caco-2, SNU-C2A (cancer) cells. **g** qRT-PCR analysis for observing effect of incorporated nanoparticles on *SOD2*, *Il-1β* and *NF-kβ* gene expression

In order for the tubular conductive THA to be applied for future bionic applications and actuation functions, it was subjected to analysis with normal colon (CCD-18 co) cells and colon cancer (Caco-2 and SNU-C2A) cell lines. The SEM analysis of the THA actuator hydrogel following treatment with cell solution was carried out to observe the presence of cells following incubation period. Figure 6a shows that the SEM images showed the presence of cells in the unwashed THA samples and the variation of pore size with the colon cancer cells demonstrating an enlarged pore morphology as compared to the normal colon cell-treated THA. This was followed by analyzing the biocompatibility of the normal and cancer colon cell lines which demonstrated the excellent biocompatibility in presence of the THA actuator hydrogel as shown from Fig. 6b with most of the cells alive in the live and dead analysis. Then, the analysis of the temperature responsive viscoelastic properties and the effect of cell treatment on the THA was carried out. The presence of elevated levels of ROS in the colon cancer cells resulted in the cleavage of the diselenide bonds in the THA which induced hydrophilicity and release of PDA affecting the elasticity and mechanical strength of the hydrogel as shown in Fig. 6c. Additionally, it also resulted in the shift of the thermoresponsive structural collapse of the polymer network demonstrating the contribution of PD@PDA toward stabilization of the THA matrix. Moreover, the resistance signals of the following treatment with normal and cancer cells demonstrated similar trends as seen with the simulated high ROS condition. The resistance of the THA as shown in Fig. 6d showed a decrease for Caco-2 and SNU-C2A cells as compared to resistance ion case of CCD-18 co-treated THA in NIR off state. When NIR was turned on the resistance of the THA specifically treated with cancer cells demonstrated a decrease owing to the NIR-selective behavior in presence of high levels of ROS. The resistance reversibility of the hydrogel was performed as shown in Fig. 6e. Moreover, for the cell-treated THA, the initial high resistance in NIR off state in the 1st cycle as compared to the later cycles may be as a result of the influence of the cell cytosol which increases the resistance to a higher value. The influence of reswelling and stabilization of the swelling rate provides uniformity to the resistance of the cell-treated THA in the successive cycles. The decrease in resistance in the presence of NIR can be attributed to the thermoresponsive behavior of the PNIPAM/PVA matrix which undergoes contraction in volume with expulsion of water. Furthermore, the decrease in resistance is higher in presence of cancer cells containing high levels of ROS like H_2_O_2_ that causes the release of PDA which absorbs NIR light in comparison to normal-cell-treated THA actuator. This was followed by the analysis of the cellular uptake of the incorporated oxidative stress-responsive nanoparticles in the THA using CLSM (Fig. S14). The THA was incubated with normal and cancer cells to observe the internalization of the nanoparticles. The presence of high levels of oxidative stress caused the uptake of ROS responsive nanoparticles in the THA by the cancer cells as seen by the regain of FL at 0.5 h and it reached a maximum at 3 h. Following 6 h of incubation the nanoparticle FL decreased as the nanoparticles escaped via lysosomal vesicles.

As an additional effect of the ROS responsive nanoparticles containing diselenide bonds and PDA, the intracellular ROS scavenging ability of the THA actuator hydrogel was analyzed. As a result, when the THA is exposed to colon cancer cells which contains high levels of intracellular ROS, the diselenide bonds in the incorporated PD@PDA are cleaved and the PDA nanoparticles are released giving rise to the antioxidant property of the THA. As shown in Fig. 6f, the red fluorescence signals of the DHE dye as it comes in contact with ROS after oxidation to 2-hydroxyethidium demonstrated a steady decrease from 1 to 12 h in case of the cancer cells showing the capability of diselenide bonds in PD@PDA to act as an antioxidant. Finally, the effect of ROS-scavenging behavior of the diselenide nanoparticles in the THA actuator hydrogel was analyzed using qRT-PCR analysis of inflammatory genes like *SOD2*, *IL-1β*, and *NF-κβ*. The effect of the ROS scavenging on redox balancing genes like *SOD2* demonstrated a decrease in the relative expression highlighting the effect of diselenide bonds in scavenging elevated ROS. Moreover, the ROS scavenging effect also influenced the downregulation of inflammatory genes like *IL-1β*, and *NF-κβ*, two important drivers of inflammation in colon cancer and other inflammatory diseases as given in Fig. 6g. Finally, the effect of the photothermal heat generation of the THA was analyzed in presence of cells to study its effect on the expression of inflammatory factors like *IL-1β* [65]. The results as shown in Fig. S15, demonstrate the slight elevation of *IL-1β* for the Caco-2 cells in presence of NIR owing to local inflammation via direct exposure to NIR as compared to only cells. Moreover, the *IL-1β* expression for Caco-2 cells with THA in presence of NIR showed a decrease in expression levels which was almost similar to that observed for Caco-2 cells with THA without the presence of NIR previously. This decrease can be primarily attributed to the ROS scavenging effect of diselenide bonds and PDA in the PD@PDA nanoparticles in the THA. Additionally, the effect of NIR and dead cells following the PTT effect of THA is not significant on the expression of *IL-1β*. Hence, the diselenide bonds therefore play an important role in scavenging elevated ROS and providing additional scavenging function in addition to its actuation functions for future bionic applications.

## Conclusion

In conclusion, an ROS- and NIR-responsive tubular conductive THA was fabricated for mimicking gastrointestinal peristalsis motion in presence of elevated oxidative stress. The actuator was fabricated using ROS responsive nanoparticle PD@PDA incorporated in a thermoresponsive polymer (PNIPAM/PVA) matrix to enable both screening and actuation functions. In the presence of high levels of ROS, the PD@PDA underwent cleavage of the diselenide bonds leading to the release of PDA which enabled selective NIR- and thermoresponsive actuation. Additionally, the released PDA effected the change of resistance and fluorescence signals alongside a shift in the elasticity and mechanical property of the THA which can be correlated with the presence of ROS in the simulated setting. Furthermore, the THA demonstrates it primary function of solid and viscous fluid transport by selectively actuating fluid such as silicon oil MR-200 and a small glass ball demonstrating its capacity for future bionic and soft robotic applications. Finally, the THA also demonstrated potent ROS-scavenging ability as displayed in presence of colon cancer cells (Caco-2 and SNU-C2A) and showed anti-inflammatory efficacy against pro-inflammatory factors like *IL-1β* and *NF-κβ*. Additionally, the THA is a unique amalgamation of an actuator and a screening platform-based on a thermoresponsive hydrogel, wherein future studies based on simultaneous treatment of the THA with gastrointestinal disease-based cells alongside ROS scavenging gastrointestinal-targeted drugs can provide an efficient drug screening platform. The presence of the drug would lead to intracellular ROS scavenging of cells thereby preventing peristalsis and changes in the resistance and FL signals in the THA and vice versa, thereby demonstrating the efficacy of the drug and its dosage. Hence, the THA provides a viable pathway for the fabrication and design of an actuation system for solid and viscous liquid transport with the potential for future bionic application and soft robotics.

## Supplementary Information

Below is the link to the electronic supplementary material.

Supplementary file1 (DOCX 29975 KB)
