# Supplementary material for: NIR and Oxidative Stress-Modulated Intestinal Peristalsis Motion of Tubular Conductive Thermo-Hydrogel Actuator
Source: Nanomicro Lett. 2026 Jul 6;18:427. doi: 10.1007/s40820-026-02257-x (PMC13338006; doi:10.1007/s40820-026-02257-x)
Supplement: Supplementary file 1 — Supplementary file1 (DOCX 29975 KB) [file 40820_2026_2257_MOESM1_ESM.docx]

**Supporting Information**

**NIR and Oxidative Stress-modulated Intestinal Peristalsis Motion of Tubular Conductive Thermo-Hydrogel Actuator**

*A Hyeon Kim^a,#^, Hayeon Jo^a,#^,* *Kaustuv Roy^a,b*^, Sung Young Park^a,b*^*

^a^Department of IT and Energy Convergence, Korea National University of Transportation, Chungju 27469, Republic of Korea

^b^School of Nanomedical Engineering, Korea National University of Transportation, Chungju 27469, Republic of Korea

***Corresponding author:**

Tel.: +82-(0)43-841-5225, Fax: +82-(0)43-841-5220, E-mail: kaustuv9323@gmail.com (Kaustuv Roy), parkchem@ut.ac.kr, (Sung Young Park).

#: A Hyeon Kim and Hayeon Jo contributed equally to this work.

**1. MATERIALS AND METHODS**

***1.1. Characterization***

A 400 MHz Joel (JNM ECZL400R) NMR (JEOL Ltd. (Japan)) spectrophotometer was used to measure the ^1^H-NMR spectra. Scanning electron microscopy (SEM, JSM-6700F, JEOL, Japan) was used to analyze the morphologies of the materials. TA instruments, DSC 2500 ((a Waters Corporation company), headquartered in New Castle, Delaware, USA), was used to assess the DSC of the THA samples (0–200°C, 10°C min^-1^, N_2_, 2 cycles). Furthermore, TA instruments, TMA 450EM ((a Waters Corporation company), headquartered in New Castle, Delaware, USA), was used to measure the thermomechanical dimension change of the THA samples (0-50^o^C, 4^o^C min^-1^, 1 Hz). The TGA analysis of the THA samples (25°C ~ 600°C (10°C min^-1^), N_2_) were measured using a TA instrument, Discovery TGA 55 instrument ((a Waters Corporation company), headquartered in New Castle, Delaware, USA). Moreover, the FT-IR of the THA samples were measured using a Shimadzu, IRXross, ATR/FT-IR spectrophotometer (Shimadzu Corporation in Kyoto, Japan). Confocal laser scanning microscopy (CLSM) images were acquired using a Nikon ECLIPSE Ti2-E confocal microscope. To assess the electrochemical properties of the materials, a sourcemeter (Keithley 2450, Tektronix, USA), and an LCR meter (4100, Wayne Kerr Electronics, UK) were employed. The rheology was measured using a HAAKE MARS modular advanced rheometer with a 40 mm parallel plate geometry. A Universal Testing Machine (UTM, SurTA 1A, Chemilab Co., South Korea) was used to measure stress-strain fracture and compressive analysis. A smartphone was utilized to present the real-time data, and an Arduino Uno microcontroller (ATmega328 P Processor) was employed as a hydrogel sensor for wireless monitoring. The Arduino Uno and smartphone were able to connect wirelessly owing to the AppGosu Bluetooth module. The photo thermal effect of the samples was analyzed by exposing the samples with 808 nm NIR laser (PSU-III-LRD) and the temperature was monitored using infra-red camera (Thermo Tracer TH9100). Fluorescence microscopy was performed using was performed using a BX53M upright microscope (OLYMPUS, South Korea) connected to a U-RFL-T power unit. For the qRT-PCR analysis, the obtained gene concentration was determined using a Nabi, UV/Vis Nano Spectrophotometer (µ2 microdigital, South Korea), while the samples were incubated using MiniAmp™ Plus Thermal Cycler (ThermoFisher, USA), and the genetic analysis was performed using QuantStudio™ 3 Real-Time PCR System, 96-well (ThermoFisher, USA).

***1.2. Effect of ROS- and NIR-responsive tubular conductive thermo-hydrogel actuator (THA) on RNA Isolation, and Quantitative Real-Time PCR (qRT-PCR)***

The treatment of Caco-2 cells at a concentration of 6 x 10^6^ cells mL^-1^, was carried out with and without THA of dimension (length: 5 mm, inner diameter: 1.5 mm, Outer diameter: 3.2 mm, wall thickness: 0.6 mm) for 12 h at 37ºC in humidified 5% CO_2_. Total RNA was extracted using RNAiso Plus reagent (#9109, Takara Bio, Japan), and complementary DNA (cDNA) was synthesized using the 5× All-In-One RT Master Mix (PrimeScript™ RT Master Mix, RR036A, Takara). Quantitative real-time PCR (qRT-PCR) was performed using TB Green® Premix Ex Taq™ II (RR830A, Takara) on a QuantStudio 3 Real-Time PCR System (ThermoFisher Scientific, USA). All reactions were carried out in triplicate. Gene expression was normalized to β-actin, and the primer sequences used were as follows: *β-actin*: Forward: 5′-GGC ATC CTC ACC CTG AAG TA-3′, Reverse: 3′-AGG TGT GGt GCC AGA TTT TC-5′, *SOD2*: Forward: 5′-CTG GAC AAA CCT CAG CCC TAA C-3′, Reverse: 3′-CAA CCA GGC TCA GGT TGT TTA A-5′, *IL-1β*: Forward: 5′-TCC AGG ATG AGG ACA TGA GCA C-3′, Reverse: 3′-GAA CGT CAC ACA CCA GCA GGT TA-5′, NF-κβ: Forward: 5′-CCT GGA TGA CTC TTG GGA AA-3′, Reverse: 3′-TCA GCC AGC TGT TTC ATG TC-5′.


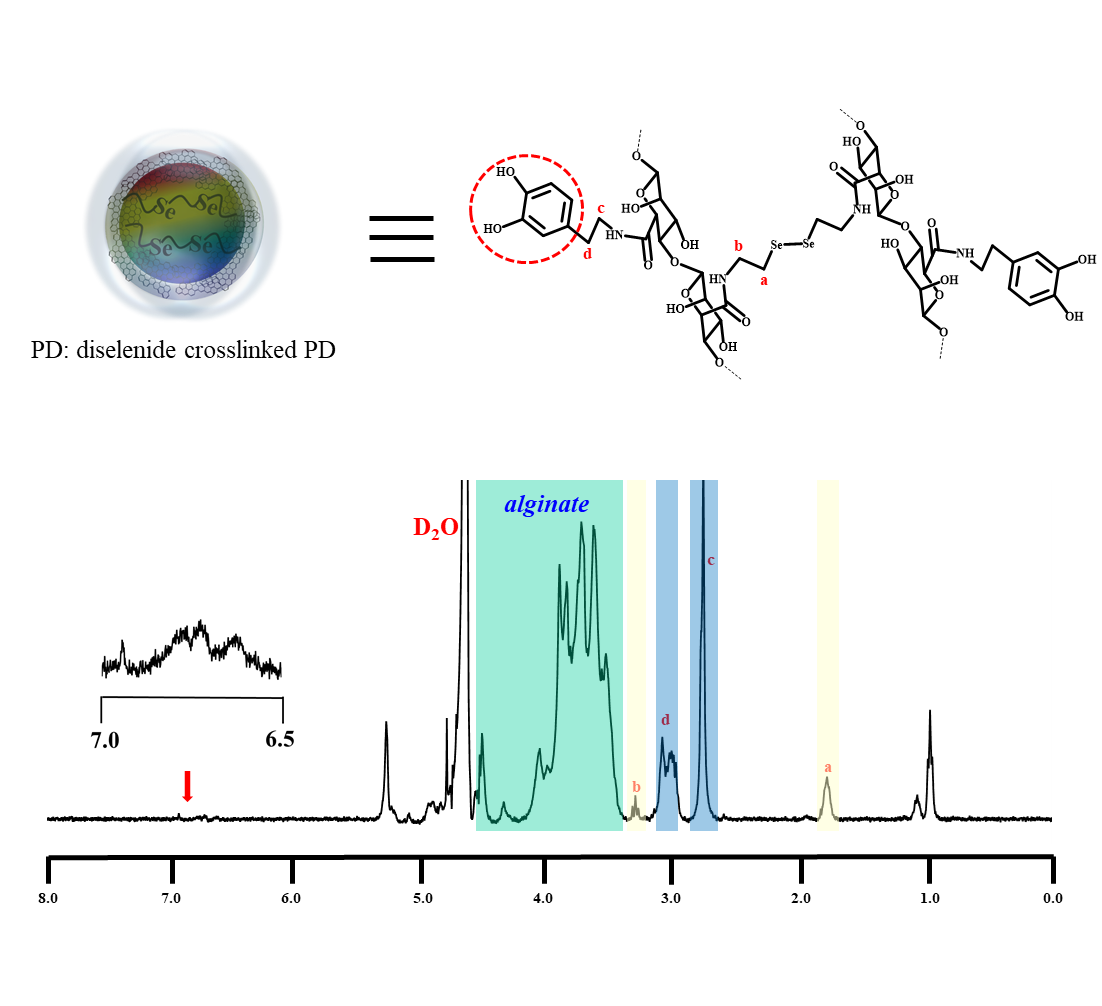


**Figure S1.** The ^1^H-NMR spectra of the diselenide crosslinked polymer dots (PD).


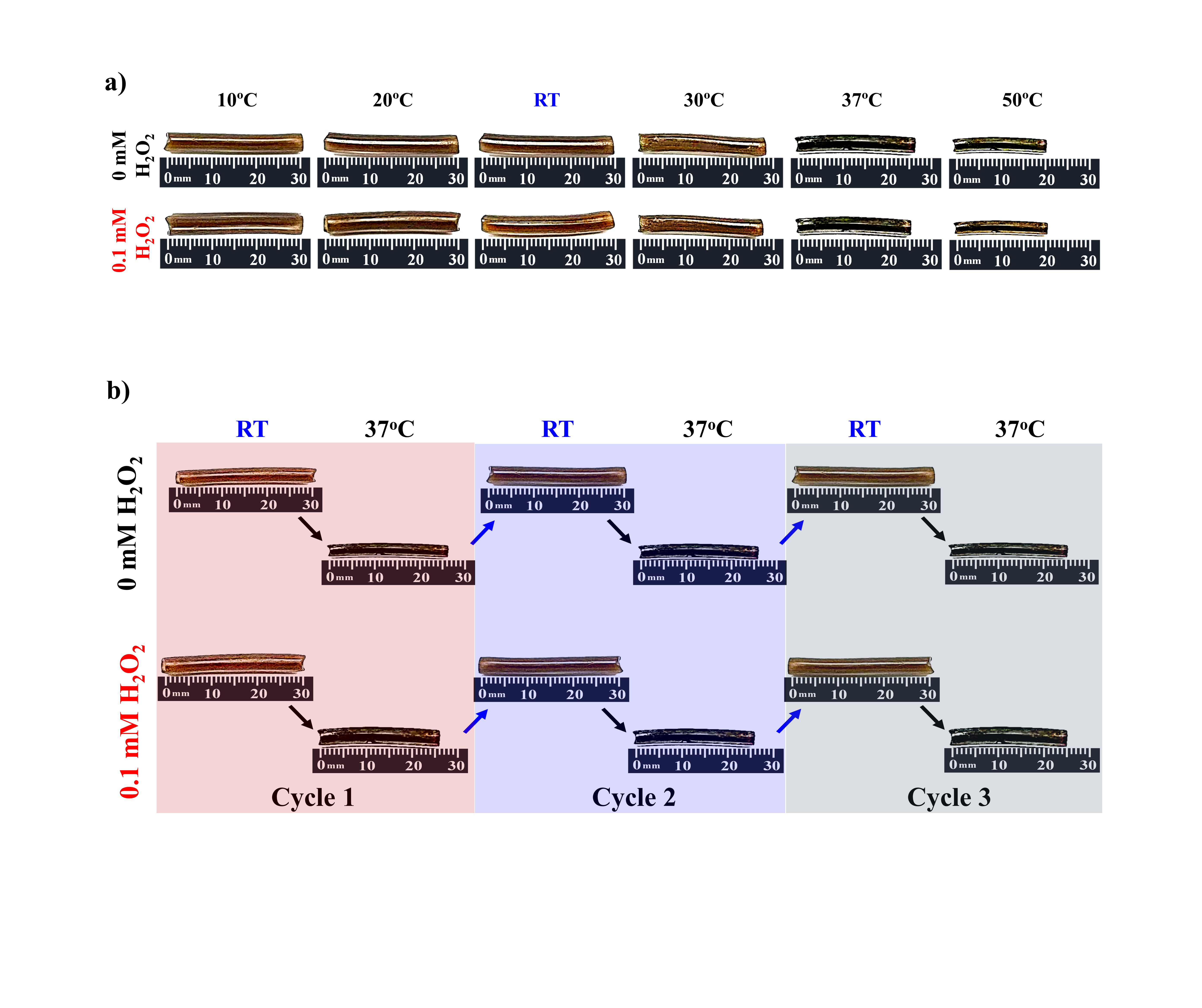


**Figure S2.** (**a**) The zoomed-in images of the THA actuator hydrogel and its thermoresponsive shrinking behavior in presence of 0 and 0.1 mM H_2_O_2_ at various temperature, and (**b**) the enlarged images of the thermoresponsive reversibility of the THA at RT and 37^o^C after treatment with 0 and 0.1 mM H_2_O_2_.


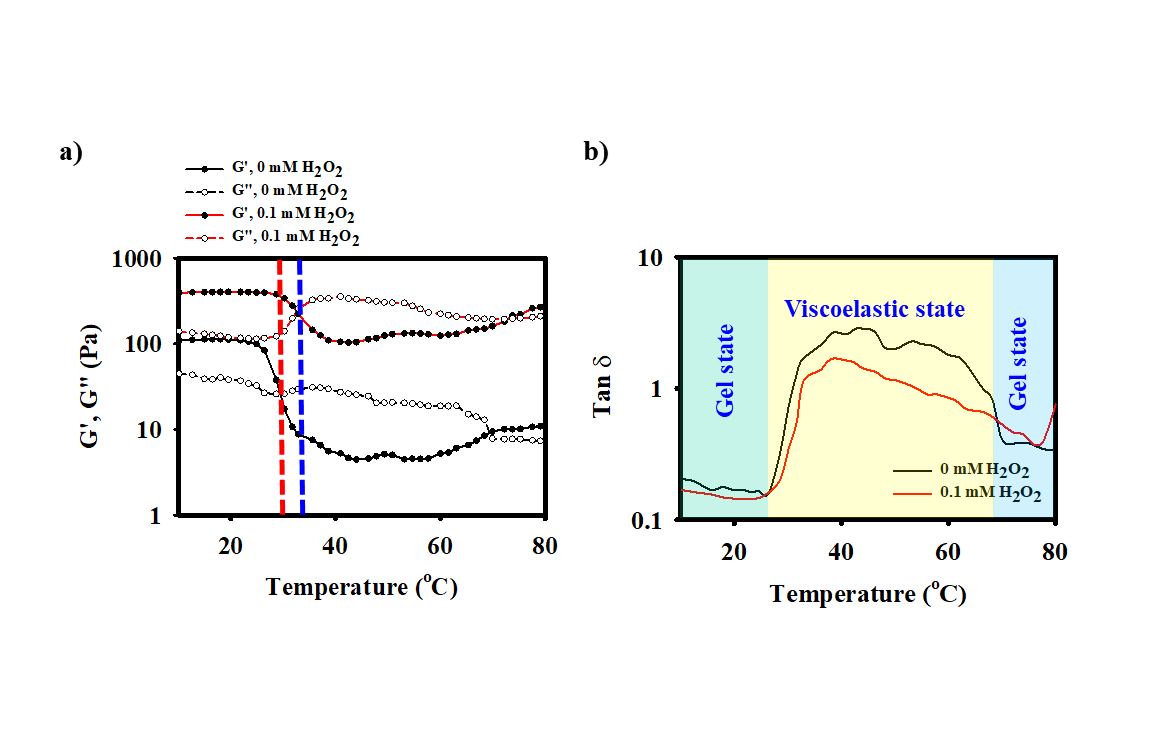


**Figure S3.** (**a**) Rheological temperature sweep of the THA at constant sheer of 10 Pa for determination of the LCST behavior, and (**b**) the Tan δ plot of the temperature sweep to show the change of phase transition at various temperatures in presence of 0 and 0.1 mM H_2_O_2_.


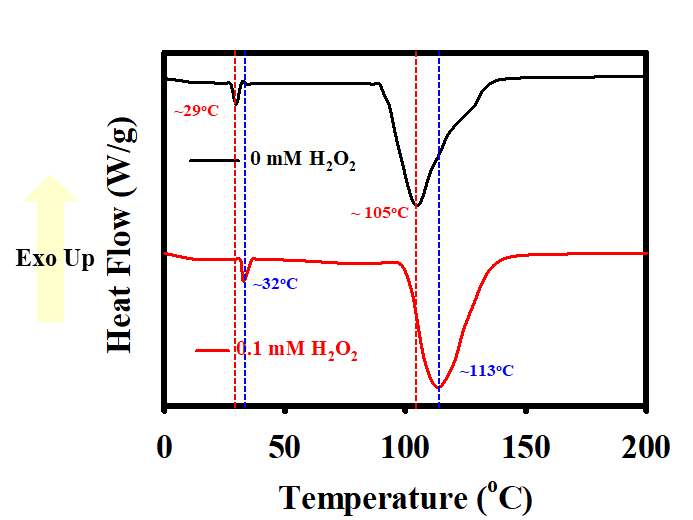


**Figure S4.** Verification of the LCST behavior of the THA actuator after treatment with 0 and 0.1 mM H_2_O_2_ *via* DSC analysis.


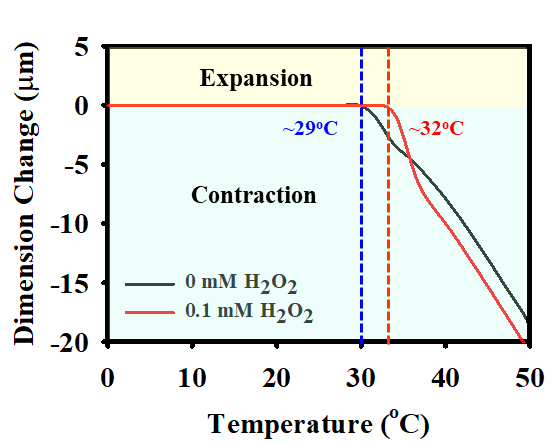


**Figure S5.** Thermomechanical analysis (TMA) of the THA actuator after treatment with 0 and 0.1 mM H_2_O_2_ for analyzing the LCST behavior and its shift.

**
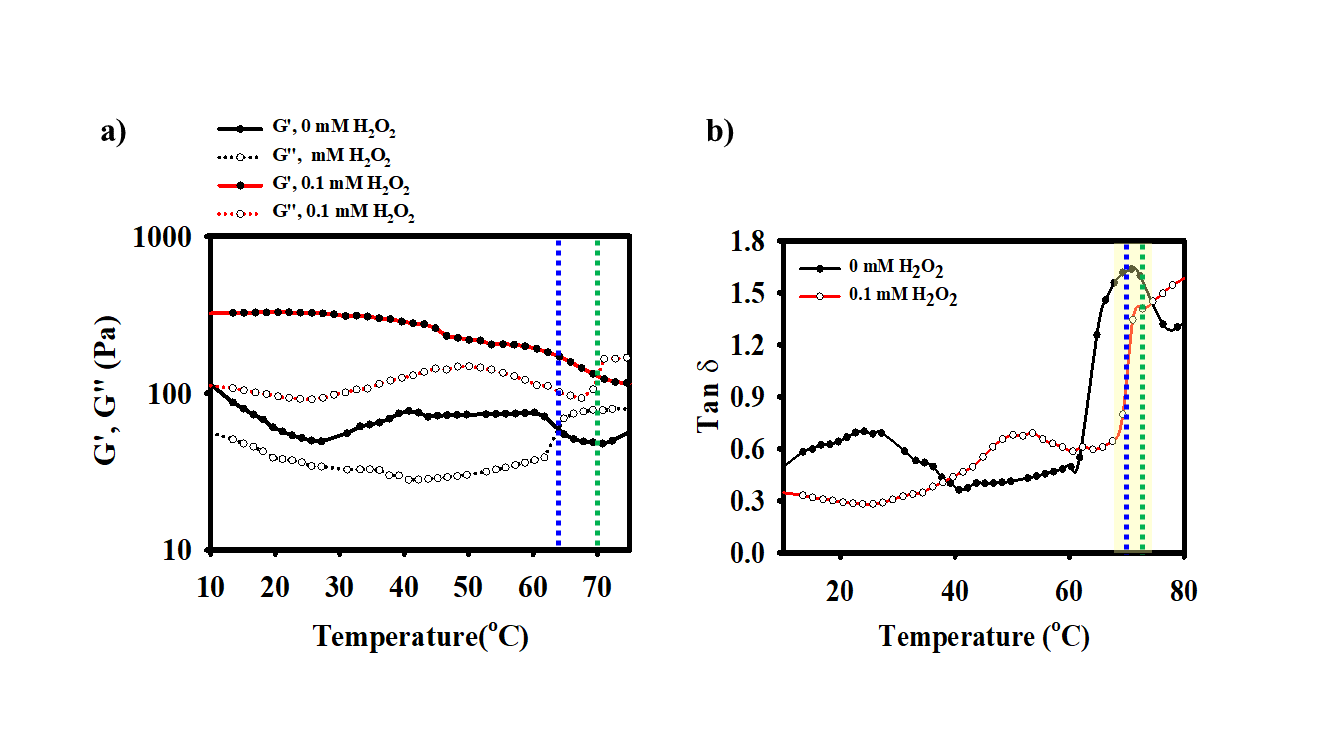
**

**Figure S6.** (**a**) Rheological temperature sweep of the THA at constant deformation of strain 0.5% and, (**b**) corresponding Tan δ plot in presence of 0 and 0.1 mM H_2_O_2_.


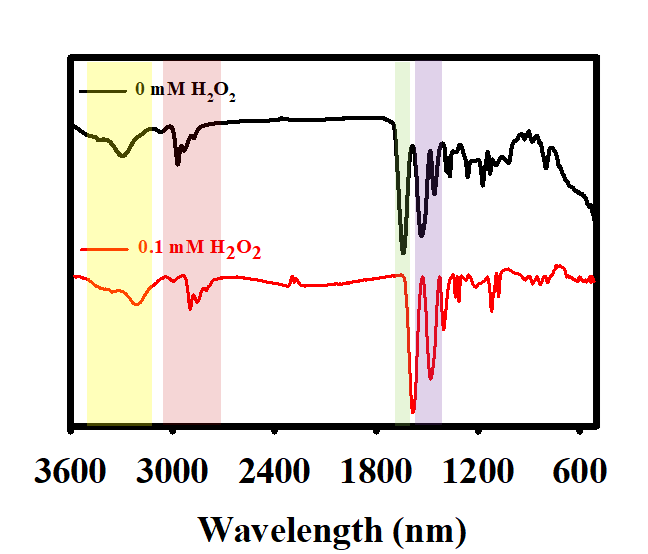


**Figure S7.** Demonstration of influence of H-bonding in the THA matrix after treatment with 0 and 0.1 mM H_2_O_2_.


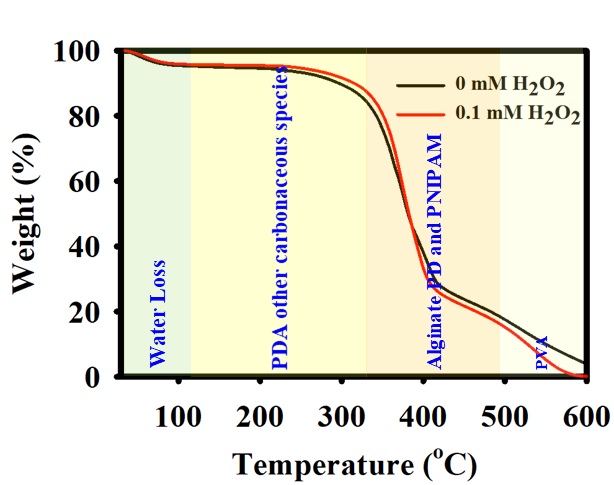


**Figure S8.** Thermo-gravimetric analysis (TGA) of the THA actuator after treatment with 0 and 0.1 mM H_2_O_2_.


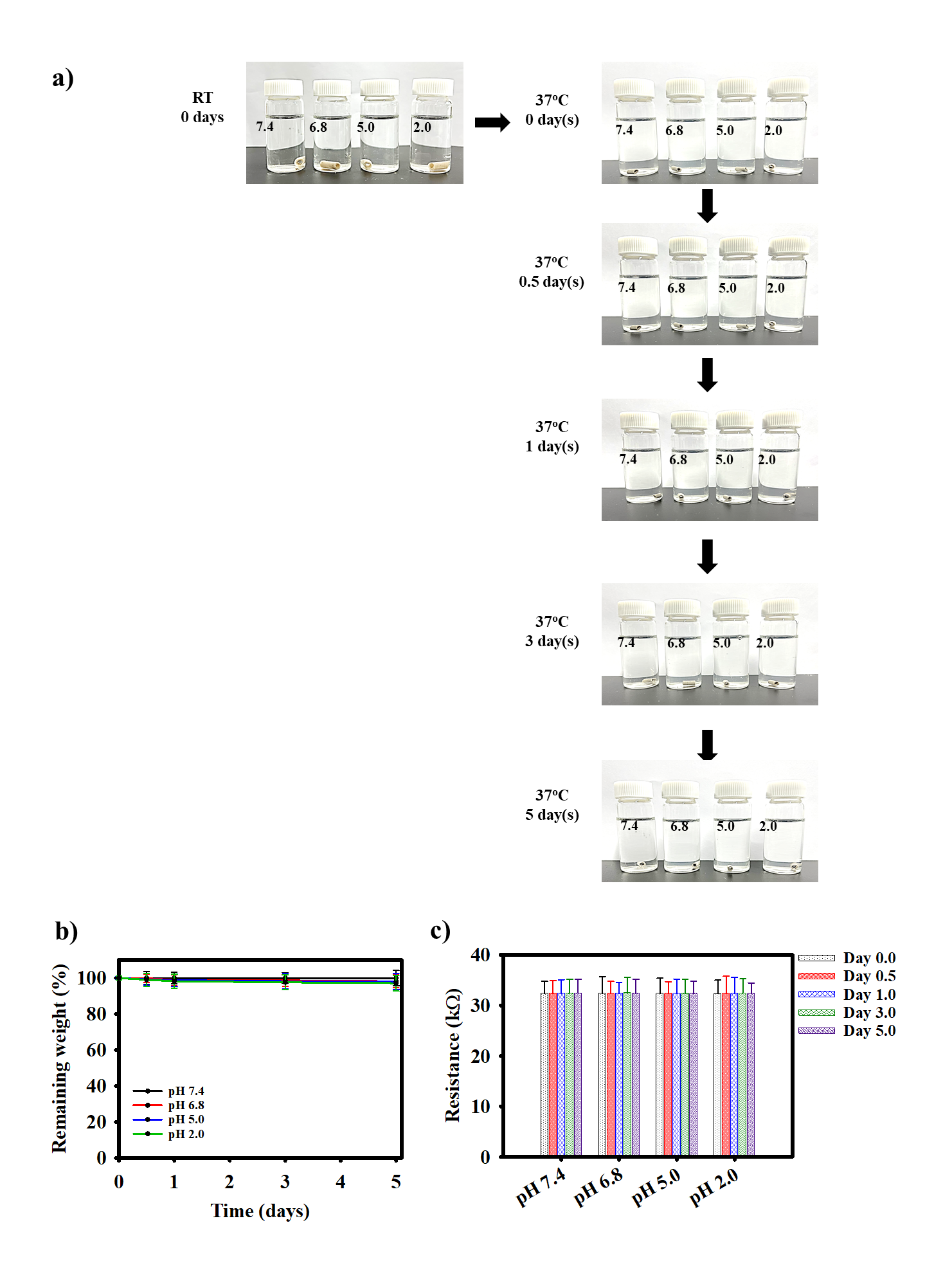


**Figure S9.**  Analysis of the overall stability of the THA actuator in presence of simulated low to extreme acidic microenvironment of the gastrointestinal system: (**a**) images of the THA at various time points, (**b**) remaining weight of the THA demonstrating its stability, and (**c**) analysis of the specificity of the actuator in presence of various pH solutions. All analysis were performed for n = 3 samples at 37^o^C.


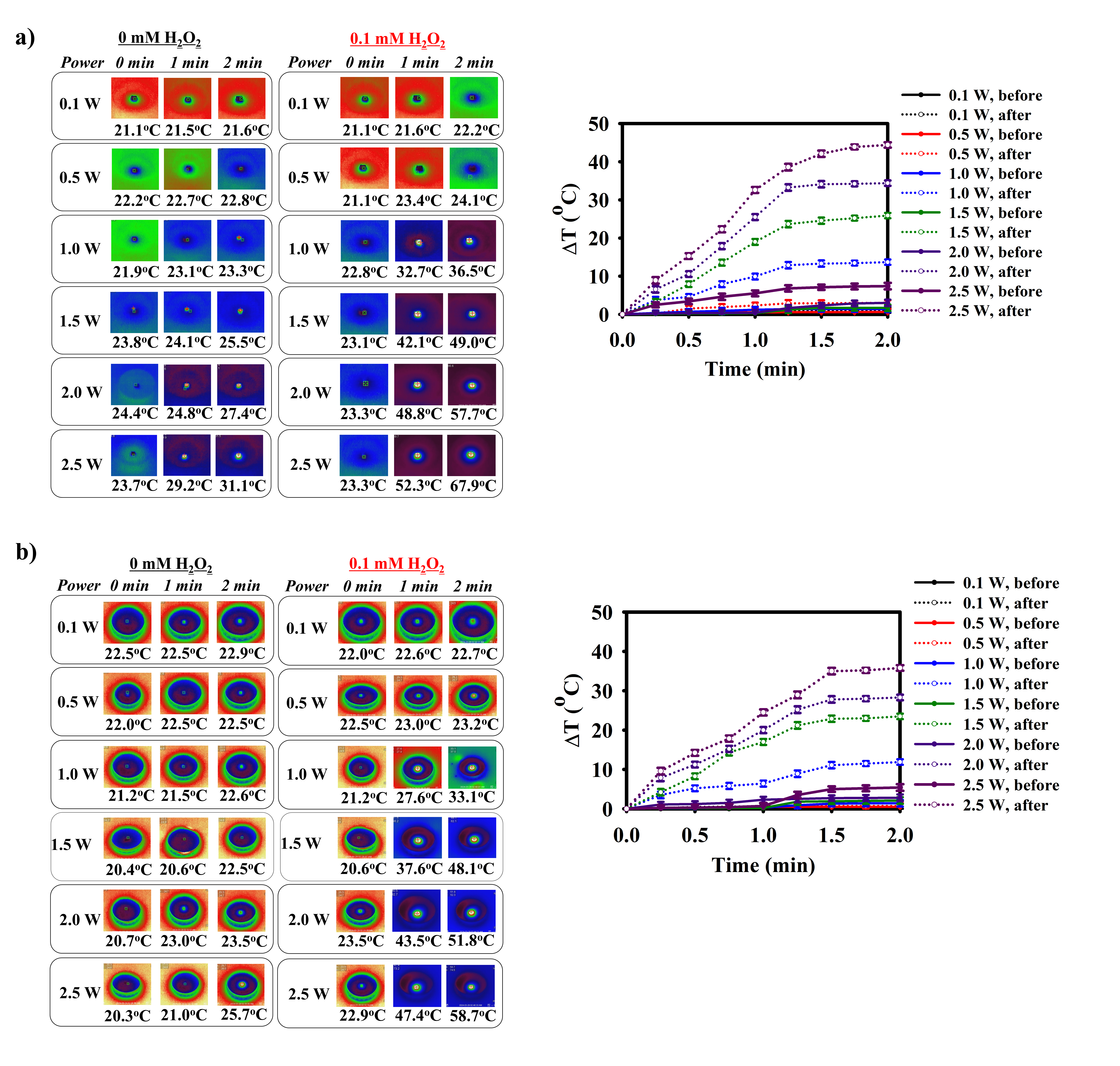


**Figure S10.** Generation of photothermal heat with variation of NIR laser power, **a)** without dipping in water, and **b)** with dipping in water after treatment of THA with 0 and 0.1 mM H_2_O_2_.

**
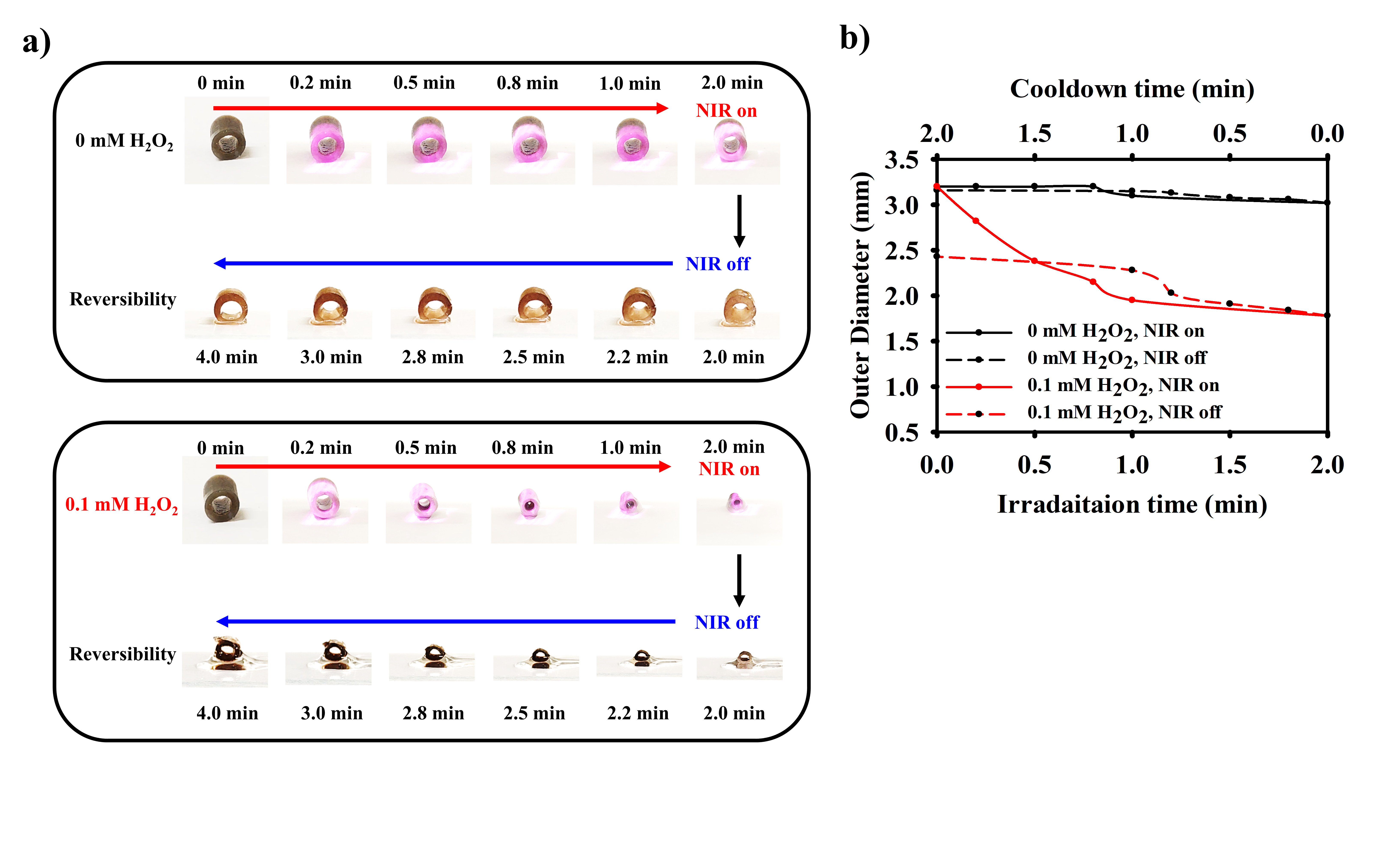
**

**Figure S11.** Change in THA diameter after exposure to NIR irradiation of 1.5 W power following, **a)** without dipping in water, and **b)** after dipping in water after treatment with 0 and 0.1 mM H_2_O_2_.


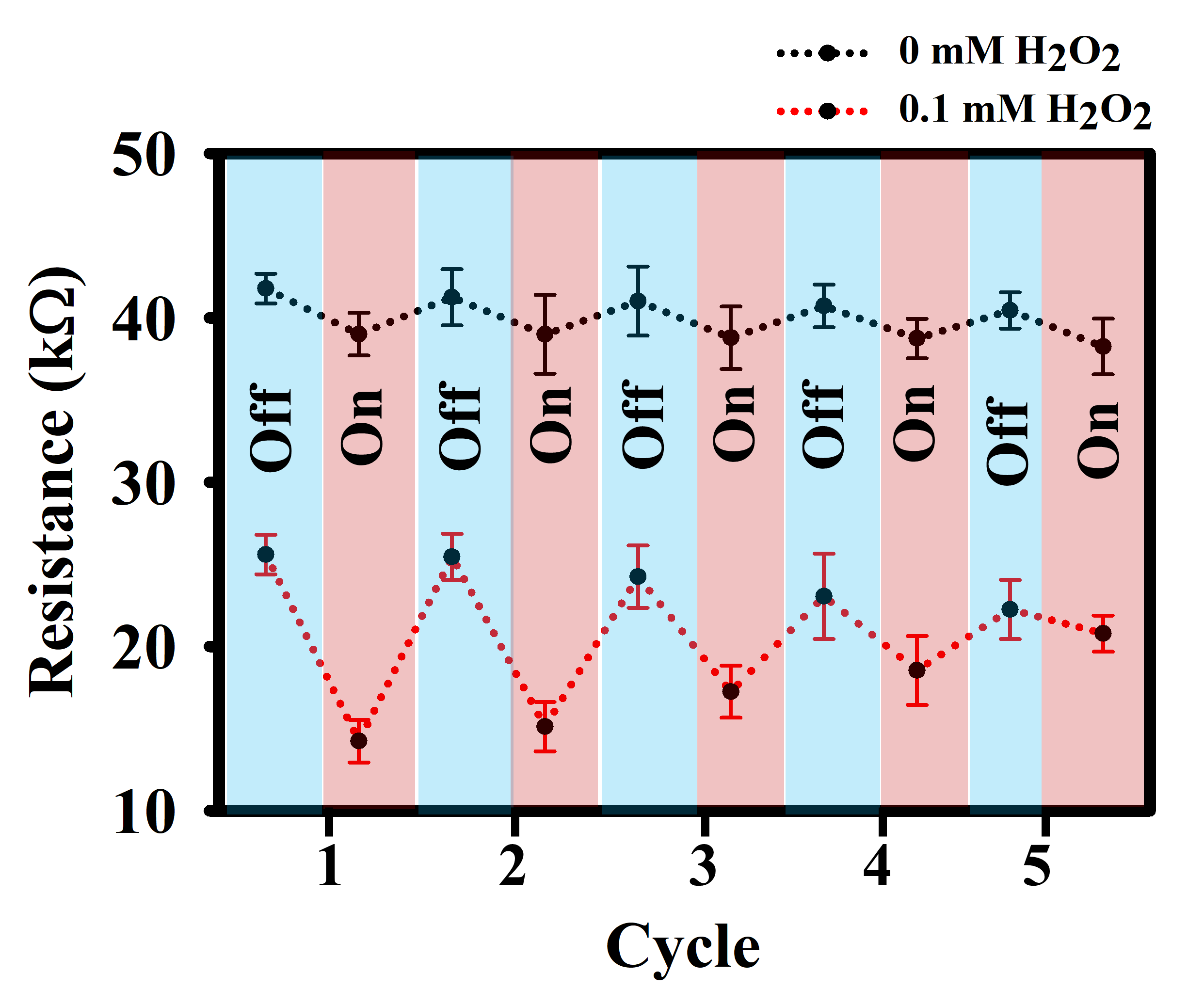


**Figure S12.** Resistance reversibility of the THA with NIR irradiation without dip in water after treatment with 0 and 0.1 mM H_2_O_2_.


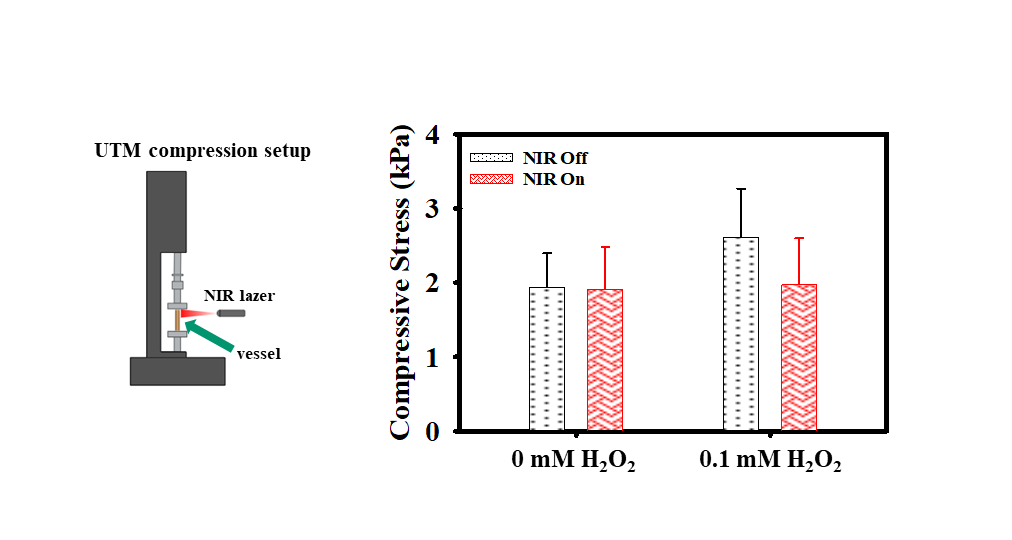


**Figure S13.** Estimation of the contractile pressure generated by the THA in the presence of an NIR laser at 1.5 W cm^-2^ power for comparison with the pressure generated during movement of a bolus through the gastrointestinal tract. All measurements were performed for n = 3 samples after treatment with 0 and 0.1 mM H_2_O_2_ for 3 h at 37^o^C.


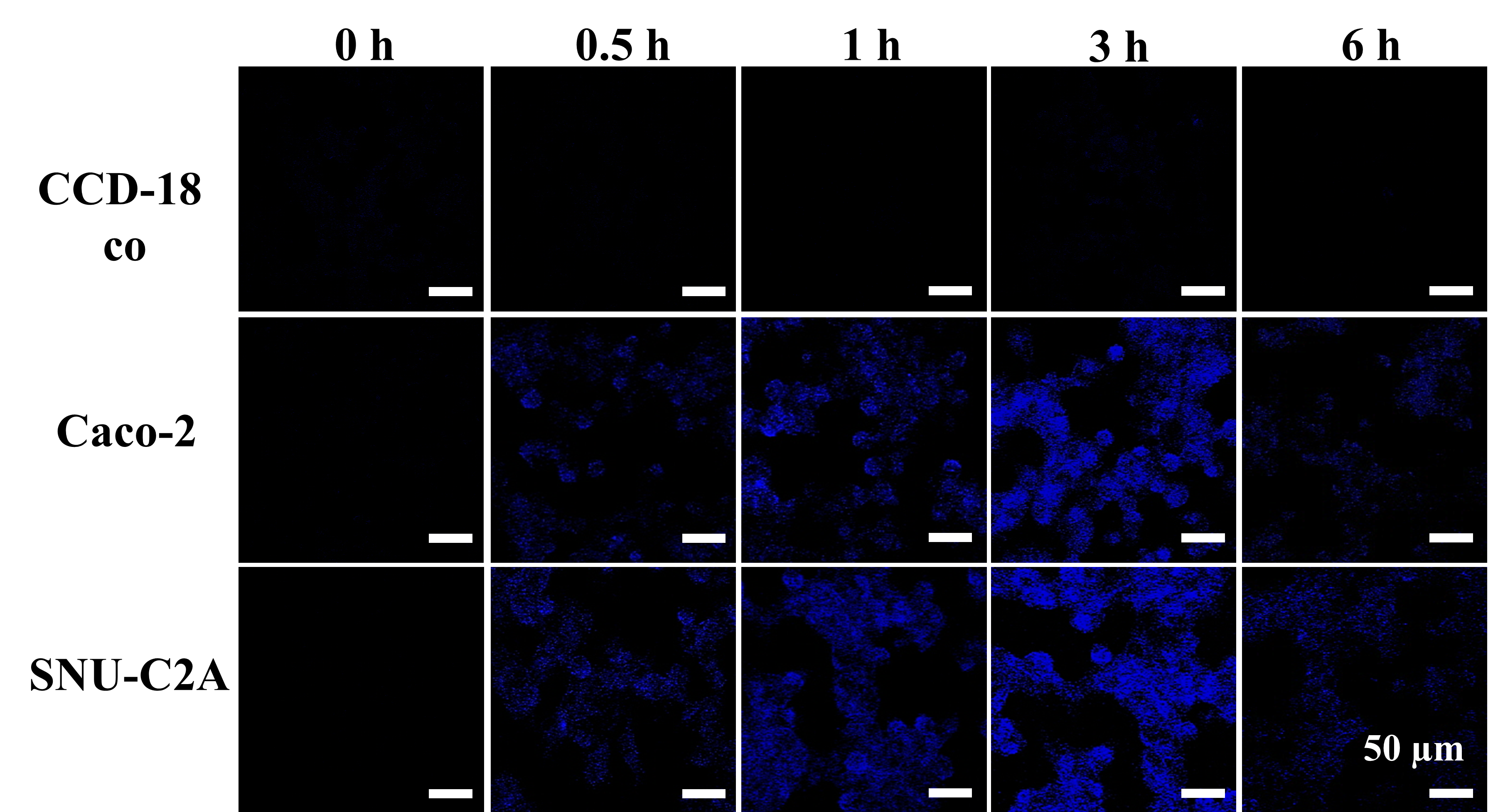


**Figure S14.** Cellular Uptake of the incorporated nanoparticles in the THA by normal and cancer cells


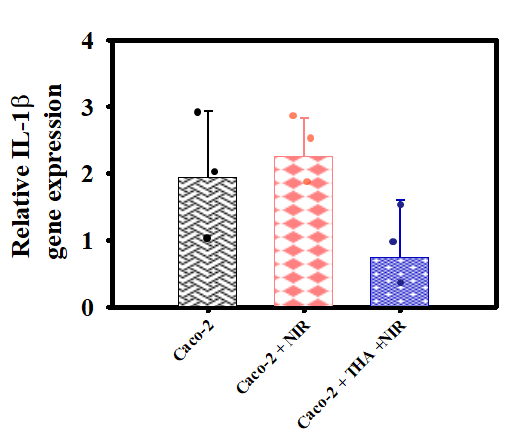


**Figure S15.** qRT-PCR analysis for analyzing the effect of NIR irradiation (for 2 min, 1.5 W cm^-2^ power) on the Caco-2 cells and Caco-2 cells treated with THA for studying the effect of cell death following PTT on expression of inflammatory factor *IL-1β* for n = 3 samples.
